# Carbon Nanocluster‐Mediated Nanoblending Assembly for Binder‐Free Energy Storage Electrodes with High Capacities and Enhanced Charge Transfer Kinetics

**DOI:** 10.1002/advs.202301248

**Published:** 2023-05-21

**Authors:** Yongkwon Song, Woojin Bae, Jeongyeon Ahn, Youhyun Son, Minseong Kwon, Cheong Hoon Kwon, Younghoon Kim, Yongmin Ko, Jinhan Cho

**Affiliations:** ^1^ Department of Chemical and Biological Engineering Korea University 145 Anam‐ro, Seongbuk‐gu Seoul 02841 Republic of Korea; ^2^ Department of Energy Resources and Chemical Engineering Kangwon National University 346 Jungang‐ro Samcheok 25913 Republic of Korea; ^3^ Department of Chemistry Kookmin University 77 Jeongneung‐ro, Seongbuk‐gu Seoul 02707 Republic of Korea; ^4^ Division of Energy Technology Materials Research Institute Daegu Gyeongbuk Institute of Science and Technology (DGIST) 333 Techno Jungang‐daero, Hyeonpung‐eup, Dalseong‐gun Daegu 42988 Republic of Korea; ^5^ KU‐KIST Graduate School of Converging Science and Technology Korea University 145 Anam‐ro, Seongbuk‐gu Seoul 02841 Republic of Korea; ^6^ Soft Hybrid Materials Research Center Advanced Materials Research Division Korea Institute of Science and Technology (KIST) 5 Hwarang‐ro 14‐gil, Seongbuk‐gu Seoul 02792 Republic of Korea

**Keywords:** binder‐free electrodes, lithium‐ion batteries, metal oxide nanoparticles, nanoblending assembly

## Abstract

The effective spatial distribution and arrangement of electrochemically active and conductive components within metal oxide nanoparticle (MO NP)‐based electrodes significantly impact their energy storage performance. Unfortunately, conventional electrode preparation processes have much difficulty addressing this issue. Herein, this work demonstrates that a unique nanoblending assembly based on favorable and direct interfacial interactions between high‐energy MO NPs and interface‐modified carbon nanoclusters (CNs) notably enhances the capacities and charge transfer kinetics of binder‐free electrodes in lithium‐ion batteries (LIBs). For this study, carboxylic acid (COOH)‐functionalized carbon nanoclusters (CCNs) are consecutively assembled with bulky ligand‐stabilized MO NPs through ligand‐exchange‐induced multidentate binding between the COOH groups of CCNs and the surface of NPs. This nanoblending assembly homogeneously distributes conductive CCNs within densely packed MO NP arrays without insulating organics (i.e., polymeric binders and/or ligands) and prevents the aggregation/segregation of electrode components, thus markedly reducing contact resistance between neighboring NPs. Furthermore, when these CCN‐mediated MO NP electrodes are formed on highly porous fibril‐type current collectors (FCCs) for LIB electrodes, they deliver outstanding areal performance, which can be further improved through simple multistacking. The findings provide a basis for better understanding the relationship between interfacial interaction/structures and charge transfer processes and for developing high‐performance energy storage electrodes.

## Graphical abstract

This study introduces an interface‐modified carbon nanocluster‐mediated nanoblending assembly that can directly and robustly bridge all interfaces between neighboring metal oxide nanoparticles (NPs) to prepare binder‐free energy storage electrodes. Based on this interfacial design, the nanoblended electrodes exhibit a homogeneously distributed structure, favorable interfacial interactions, fast charge transfer kinetics, and exceptional performance in lithium‐ion batteries (LIBs).

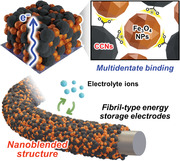

## 1 Introduction

To meet the rapidly growing demand for modern portable/wearable electronics, energy storage applications, such as lithium‐ion batteries (LIBs), require higher energy/power densities and better operational stabilities with smaller and lighter electrodes than the current ones.^[^ ^1^, ^2^, ^3^, ^4^, ^5^, ^6^, ^7^, ^8^, ^9^ ^]^ To achieve these goals, many research efforts have focused on improving capacities and rate capabilities through the synthesis of novel electrode materials and/or the rational design of electrode structures.^[^ ^5^, ^6^, ^7^, ^8^, ^9^ ^]^ Recently, areal performance parameters, such as areal capacity, have been recognized as another important factors reflecting the practical levels of electrodes.^[^ ^10^, ^11^, ^12^ ^]^ As a promising approach for enhancing this areal performance, highly porous and conductive fibril‐type textiles have attracted much attention as three‐dimensional (3D) current collectors due to their large specific surface area for high mass loading of active components.^[^ ^13^, ^14^, ^15^ ^]^ That is, blended slurries of electrode components, which are typically composed of powder‐type active materials (e.g., metal oxides (MOs)), conductive carbon additives (e.g., carbon blacks (CBs) and carbon nanotubes (CNTs)), and polymeric binders,^[^ ^16^ ^]^ are deposited on porous fibril‐type current collectors (FCCs) using conventional coating methods, including dip coating, doctor‐blade coating, printing, and vacuum filtration.^[^ ^17^, ^18^, ^19^, ^20^ ^]^ Although these approaches have contributed to some improvements in the areal performance of electrodes, there are still critical issues that need to be addressed in order to further improve energy storage performance.

Previously reported studies have not fully considered the influence of complementary interfacial interactions among slurry components and uniformity of blended components on the overall performance of electrodes. For example, when preparing MO nanoparticle (NP)‐based slurries, hydrophobic carbon additives (such as CBs or CNTs) are mechanically blended with active MO NPs with the aid of polymeric binders.^[^ ^16^, ^21^ ^]^ However, unfavorable interfacial interactions between MO NPs and carbon additives, between MO NPs and polymeric binders, and between neighboring MO NPs induce aggregated and segregated phases.^[^ ^22^ ^]^ In addition, the slurry‐coated electrodes unavoidably accompany the heterogeneous migration of components (i.e., low‐density carbon additives float to the top surface, whereas high‐density MO NPs sink to the bottom) during solvent evaporation.^[^ ^12^, ^23^ ^]^ These phenomena lead to the uneven distribution of conductive components among MO NPs, hindering the formation of efficient charge transfer pathways within electrodes. Furthermore, weakly adsorbed components are highly vulnerable to the large volume expansion of MO NPs during lithiation in rechargeable LIBs, resulting in the structural failure and rapid capacity fading of electrodes.^[^ ^24^ ^]^ Although these drawbacks can be alleviated through the use of polymeric binders,^[^ ^25^ ^]^ insulating/inactive polymeric binders act as contact resistance sites, thereby reducing the energy efficiencies of electrodes.^[^ ^26^ ^]^ Besides, when porous FCCs are utilized as 3D current collectors, conventional coating methods have much trouble uniformly depositing highly concentrated and viscous slurries on entire regions ranging from the exterior to the interior of the FCCs.^[^ ^27^ ^]^ In this case, the aggregation and segregation of electrode components due to unfavorable interfacial interactions can block the numerous voids of the porous FCCs.

Particularly, when using high‐energy MO NPs with poor electrical conductivities, the nonhomogeneous blending‐induced aggregation severely impedes electron transfer and ion diffusion, decreasing the active surface area and increasing the total resistance of electrodes.^[^ ^28^ ^]^ Although the incorporation of one‐dimensional CNTs enables the formation of a nanoporous conducting network‐structure within the MO NP arrays, their bulky size and low tap densities significantly decrease the volumetric performance of electrodes.^[^ ^29^ ^]^ An alternative approach for improving the packing densities (or volumetric performance) is to use small‐sized carbon nanoclusters (CNs), which are composed of nanosized CBs, instead of bulky CNTs. However, the hydrophobic CNs tend to be easily aggregated,^[^ ^30^ ^]^ resulting in the high contact resistance at the interfaces between neighboring MO NPs that are free of CNs. Despite these critical issues, the effects of the physical size and interfacial interactions of carbon additives on energy storage performance have been overlooked in the last few decades and less studied than other components in MO NP‐based electrodes.

Thus, beyond the simple incorporation of conductive components, the effective spatial distribution/arrangement characteristics of electrode components based on favorable interfacial interactions should be designed to fully exploit the large active surface area and high energy capacities of nanosized MOs for enhancing overall performance levels. That is, developing a direct nanoblending assembly of active MO NPs and conductive CNs, which allows robust interfacial interactions and conformal coating without the use of insulating polymeric binders, can underpin the successful development of high‐performance energy storage electrodes.

**Scheme 1:**
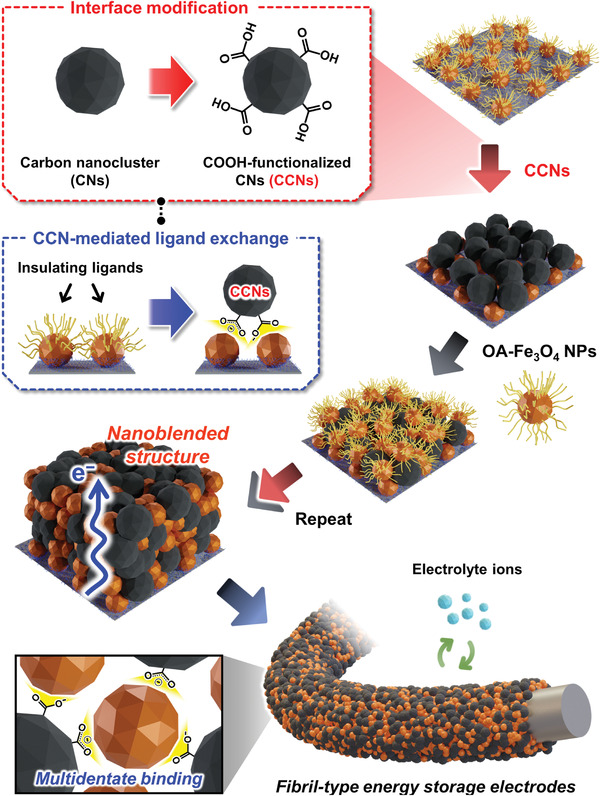
Schematic illustrations of a carboxylic acid (COOH)‐functionalized carbon nanoclusters (CCN)‐mediated nanoblending assembly based on ligand‐exchange reaction‐induced multidentate binding to prepare binder‐free Fe_3_O_4_ NP‐based energy storage electrodes.

In this study, we introduce an interface‐modified CCN‐mediated nanoblending assembly to prepare binder‐free MO NP electrodes with a homogeneously distributed structure, favorable interfacial interactions, and remarkably high energy storage performance in LIBs. We highlight that our approach is also effectively applicable to porous FCCs, producing textile‐type LIB electrodes with high areal performance. For this study, carboxylic acid (COOH) groups are introduced on the surface of hydrophobic CNs; then, the COOH‐functionalized CNs (CCNs) are used as conductive linkers to bridge all interfaces directly and robustly between adjacent MO NPs. Specifically, the CCNs are consecutively layer‐by‐layer (LbL)‐assembled with bulky oleic acid (OA)/oleylamine (OAm) ligand‐stabilized Fe_3_O_4_ NPs (denoted as OA‐Fe_3_O_4_ NPs) through ligand‐exchange‐induced multidentate binding between the COOH groups of CCNs and the surface of NPs (**Scheme** **1**). Additionally, those oxygen‐containing functional groups on the CCN surface not only induce stable interfacial interactions but also provide additional capacity through reversible redox reactions with Li ions. Importantly, our approach can be widely applied to various high‐energy MO NPs such as OA‐MnO NPs, OA‐TiO_2_ NPs, and OAm‐ITO NPs well as OA‐Fe_3_O_4_ NPs using the same favorable interfacial interactions with CCNs. Based on the formation of efficient charge transfer pathways, the CCN‐mediated Fe_3_O_4_ NP electrodes show high capacities, good rate capabilities, and long‐term operational stabilities in LIB systems. Furthermore, when using 3D porous FCCs, the formed electrodes deliver a considerably enhanced areal capacity of ≈5.67 mAh cm^−2^, which can be further increased up to ≈11.0 mAh cm^−2^ through the additional multistacking of the electrodes. To our knowledge, this CCN‐mediated nanoblending assembly has been never reported to date, and the obtained performance from this assembly approach surpasses that of previously reported MO‐based LIB electrodes. Furthermore, our approach can give insight into the effects of interfacial interaction/structures on charge transfer behaviors and provide a basis for developing a variety of other high‐performance energy storage devices as well as LIBs.

## 2 Results and Discussion

### 2.1 Preparation of CCN‐Mediated Fe_3_O_4_ NP Electrodes

**Figure 1:**
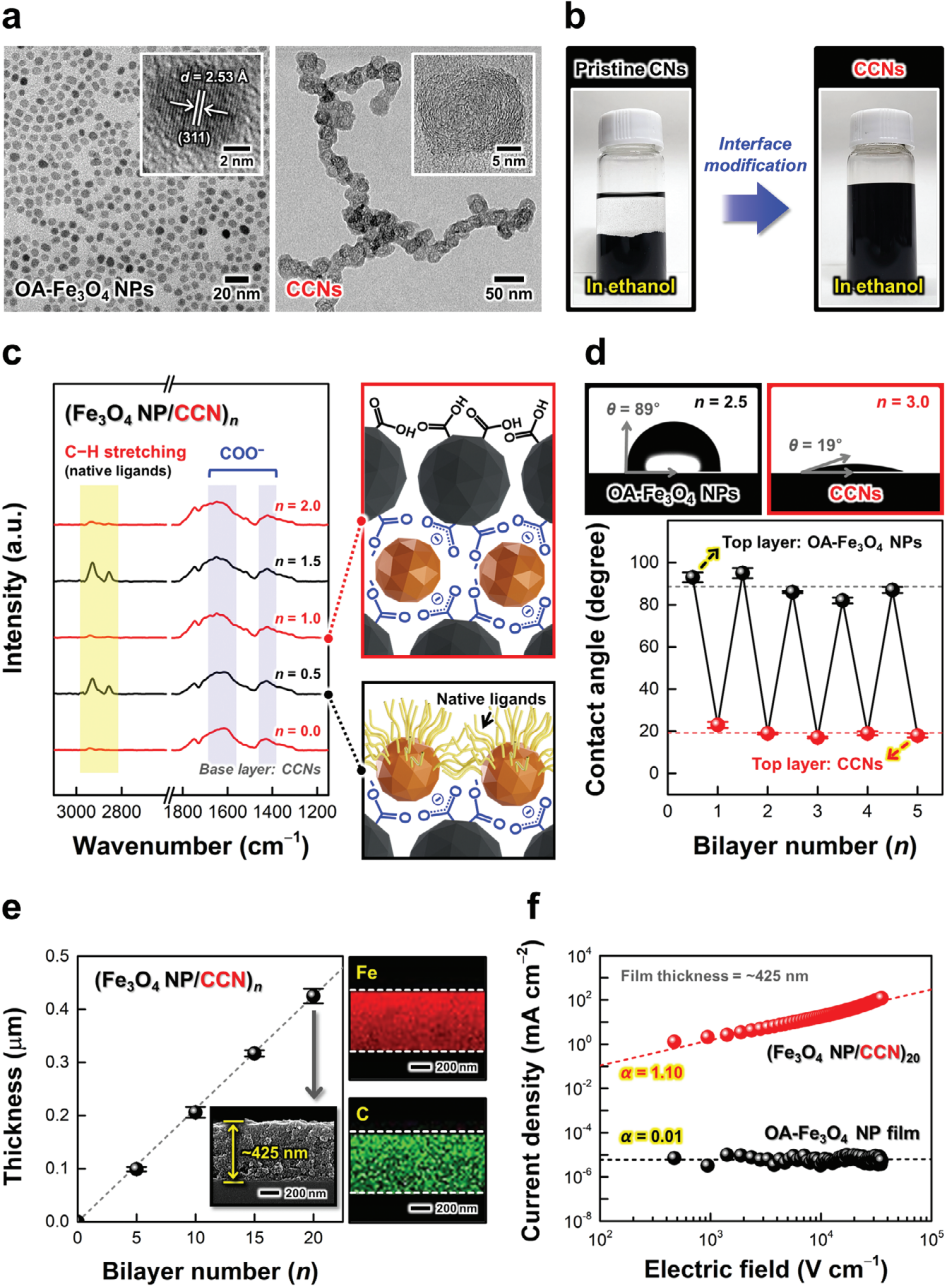
a) High‐resolution transmission electron microscopy (HR–TEM) images of OA‐Fe_3_O_4_ nanoparticles (NPs) (left) and carboxylic acid (COOH)‐functionalized carbon nanoclusters (CCNs) (right). The two insets show a highly ordered crystal structure of ≈7 nm‐sized OA‐Fe_3_O_4_ NPs with a lattice fringe spacing (*d*) of ≈2.53 Å ascribed to the (311) phase and a paracrystalline structure of ≈20 nm‐sized CCNs. b) Photographs of hydrophobic CNs (left) and surface‐modified hydrophilic CCNs (right) in ethanol. c) Bilayer number‐dependent Fourier transform infrared (FTIR) spectra and schematic diagrams during the LbL assembly of (Fe_3_O_4_ NP/CCN)*_n_* composites. d) Photographs of sessile water droplets at *n* = 2.5 with the outermost OA‐Fe_3_O_4_ NP layer (top left) and *n* = 3.0 with the outermost CCN layer (top right). Water contact angle changes (bottom) of (Fe_3_O_4_ NP/CCN)*_n_* composites as a function of bilayer number. e) Film thickness changes of (Fe_3_O_4_ NP/CCN)*_n_* composites as a function of bilayer number, along with cross‐sectional field‐emission scanning electron microscopy (FE–SEM) and energy‐dispersive X‐ray spectroscopy (EDS) elemental mapping images at *n* = 20. f) Current density (log *J*)–electric field (log *E*) plots of (Fe_3_O_4_ NP/CCN)_20_ composites under the external voltage range of ±1.5 V.

To prepare high‐performance energy storage electrodes with high capacities and excellent rate capabilities, we first synthesized toluene‐dispersible OA‐Fe_3_O_4_ NPs with a diameter of ≈7 nm and ethanol‐dispersible CCNs (the CCNs were composed of dozens of approximately 20 nm‐sized CBs) through the surface modification of hydrophobic CNs, as confirmed through high‐resolution transmission electron microscopy (HR–TEM) and X‐ray diffraction (XRD) (**Figure** **1**a,b; Figure S1, Supporting Information). The CCNs exhibited two broad diffraction peaks at ≈24.6° and 43.6°, which originated from the (002) and (100) planes of amorphous graphitic phases, respectively, coinciding with those of pristine CNs.^[^ ^31^ ^]^ Fourier transform infrared (FTIR) spectra of CCNs showed strong absorption peaks at 1712 cm^−1^ (by C=O stretching) and at 1242 cm^−1^ (by C−O stretching), clearly implying the formation of COOH groups on the CCN surface. (Figure S2, Supporting Information).^[^ ^32^ ^]^ In addition, the C−C stretching peak (at 1516 cm^−1^) associated with the aromatic rings of CCNs was almost identical to that of pristine CNs,^[^ ^33^ ^]^ indicating that their original chemical structures (or physical properties) were retained even after surface modification. When these CCNs were spin‐coated with a thickness of ≈420 nm on SiO_2_/Si wafers, the formed CCN films displayed an electrical conductivity of ≈1.97 S cm^−1^ and a sheet resistance of ≈1.21 × 10^4^ Ω sq^−1^, suggesting that the surface‐modified CCNs could effectively operate as conductive components within electrodes. Furthermore, the hydrophilic COOH groups on the CCN surface allowed the good dispersion stability of CCNs in ethanol (Figure 1b).

A notable characteristic of CCNs is that they can be sequentially assembled with OA‐Fe_3_O_4_ NPs through an in situ ligand‐exchange reaction between the COOH groups of CCNs and the bulky native ligands (i.e., OA and OAm) bound to the NP surface. To better understand this ligand‐exchange reaction, bilayer number (*n*)‐dependent FTIR spectra were investigated during the sequential LbL depositions of OA‐Fe_3_O_4_ NPs and CCNs using an attenuated total reflection (ATR) mode (Figure 1c; Figure S3, Supporting Information). In this case, OA‐Fe_3_O_4_ NPs exhibited strong C−H stretching peaks (at 3000−2800 cm^−1^) that were assigned to the long alkyl chains of native OA/OAm ligands,^[^ ^34^ ^]^ whereas no notable absorption peaks were identified for CCNs in the same region (Figure S4, Supporting Information). Additionally, the other characteristic absorption peaks of CCNs (i.e., C=O, C−C, and C−O stretching) almost overlapped with the peaks originating from the functional moieties of OA/OAm ligands on the surface of Fe_3_O_4_ NPs.^[^ ^32^, ^33^ ^]^ Therefore, the ligand replacement behaviors between the native OA/OAm ligands and the CCNs were confirmed by monitoring the intensity changes in C−H stretching vibrations at 3000−2800 cm^−1^. When OA‐Fe_3_O_4_ NPs were deposited on the CCN‐coated substrate (see *n* = 0.5 in Figure 1c), the C−H stretching peaks from OA/OAm ligands bound to the top surface of NPs obviously appeared. However, these peaks disappeared by sequentially depositing CCNs on the outermost Fe_3_O_4_ NP layer (see *n* = 1 in Figure 1c). Since the C−H stretching peaks were derived only from OA/OAm ligands, the periodic changes in the peak intensities according to the alternating depositions of OA‐Fe_3_O_4_ NP and CCN layers suggested that the bulky/insulating OA/OAm ligands were effectively replaced by the CCNs during the LbL assembly (see *n* = 1.5 and 2.0 in Figure 1c). Particularly, when the CCNs were assembled with OA‐Fe_3_O_4_ NPs, the COOH groups on the surface of CCNs were converted to anionic carboxylate ion (COO^−^) groups, forming strong binding between one or two O atoms and Fe atoms.^[^ ^35^, ^36^ ^]^ That is, CCNs with multiple COOH groups acted as multidentate ligands with higher interfacial affinities (i.e., adsorption energy) for the surface of NPs than mono‐/bidentate OA and monodentate OAm ligands,^[^ ^37^ ^]^ effectively removing the bulky native ligands. These phenomena demonstrated that the CCNs could directly bridge all interfaces between adjacent Fe_3_O_4_ NPs through ligand‐exchange reaction‐induced multidentate binding without the use of insulating polymeric binders.

This ligand‐exchange reaction could be further confirmed by the water contact angle measurements of (Fe_3_O_4_ NP/CCN)*_n_* composites with different layer numbers (Figure 1d). When an OA‐Fe_3_O_4_ NP layer was deposited on the substrate, the contact angle was measured as ≈89 ± 5°, representing hydrophobic surface properties due to the residual native ligands on the top surface of NPs. However, when a hydrophilic CCN layer was additionally deposited on the OA‐Fe_3_O_4_ NP layer‐coated substrate, the contact angle significantly reduced to ≈19 ± 2°. These dramatic changes in the contact angles were periodically observed as the outermost layers changed from OA‐Fe_3_O_4_ NPs to CCNs or vice versa, clearly suggesting that the hydrophobic native ligands on the NP surface were effectively removed by the hydrophilic CCNs during the LbL assembly. This hydrophilic surface chemistry indicated that our CCN‐mediated Fe_3_O_4_ NP electrodes possessed excellent interfacial wettability to polar electrolytes and thereby induced fast and efficient electrochemical reactions (the more details are given later).^[^ ^28^ ^]^

The vertical growth behaviors of the (Fe_3_O_4_ NP/CCN)*_n_* composites were investigated through UV−Vis spectroscopy and quartz crystal microbalance (QCM). As shown in Figure S5 (Supporting Information), the overall absorbance regularly increased as the bilayer number increased, which was consistent with the QCM analyses (Figure S6, Supporting Information). In this case, the average mass changes (Δ*m*) for the depositions of OA‐Fe_3_O_4_ NPs and CCNs, obtained from the frequency changes (−Δ*F*) using the Sauerbrey equation (see the Experimental Section in Supporting Information), were measured to be ≈4.95 ± 0.2 and 0.75 ± 0.2 µg cm^−2^ per layer, respectively. Field‐emission scanning electron microscopy (FE–SEM) and energy‐dispersive X‐ray spectroscopy (EDS) elemental mapping images revealed that the total film thicknesses of the (Fe_3_O_4_ NP/CCN)*_n_* composites increased linearly from ≈99 to ≈425 nm with increasing bilayer number from 5 to 20, showing the uniform distribution of CCNs and Fe_3_O_4_ NPs within the composites (Figure 1e; Figure S7, Supporting Information). Furthermore, the HR–TEM image of the (Fe_3_O_4_ NP/CCN)_20_ composites displayed the formation of intimate interphases between Fe_3_O_4_ NPs and CCNs (Figure S8, Supporting Information). The (Fe_3_O_4_ NP/CCN)_20_ composites exhibited a higher mass density of ≈2.68 g cm^−3^ than those of previously reported slurry‐coated FeO*_x_* electrodes (<1.90 g cm^−3^).^[^ ^38^, ^39^, ^40^ ^]^ Therefore, these results proved that our CCN‐mediated nanoblending assembly generated a highly homogeneous and densely NP‐packed structure with precisely controlled mass loading (or film thickness). In addition, we confirmed that this approach using CCNs could be applied to various high‐energy MO NPs such as OA‐MnO NPs, OA‐TiO_2_ NPs, and OAm‐ITO NPs well as OA‐Fe_3_O_4_ NPs, for preparing the composite films (Figure S9, Supporting Information).

We examined the electrical properties of the (Fe_3_O_4_ NP/CCN)_20_ composites to verify the roles of CCNs as conductive linkers (Figure 1f; Figure S10, Supporting Information). In this case, the CCN‐mediated Fe_3_O_4_ NP films exhibited high current levels (≈10^−1^ mA) with ohmic conduction behaviors (*α* = 1.10) in the relationship between current density (*J*) and electric field (*E*) [*J* ∝ *E* ^*α*^];^[^ ^41^ ^]^ this result was in stark contrast to the pristine OA‐Fe_3_O_4_ NP film showing insulating properties (current levels of ≈10^−8^ mA with *α* = 0.01). These improvements in the electrical properties of the CCN‐mediated films occurred mainly due to the formation of a uniformly nanoblended/interconnected structure and the effective removal of insulating native ligands by conductive CCNs during the LbL assembly, which markedly reduced the contact resistance at the interfaces between adjacent Fe_3_O_4_ NPs. That is, the abovementioned results clearly suggested the possibility that the CCN‐mediated Fe_3_O_4_ NP composites could be used as electrochemically active electrodes with the attractive characteristics of excellent electrolyte wettability, controllable mass loading (or film thickness), and good charge transfer kinetics.

### 2.2 LIB Electrodes Using a CCN‐Mediated Fe_3_O_4_ NP Assembly

**Figure 2:**
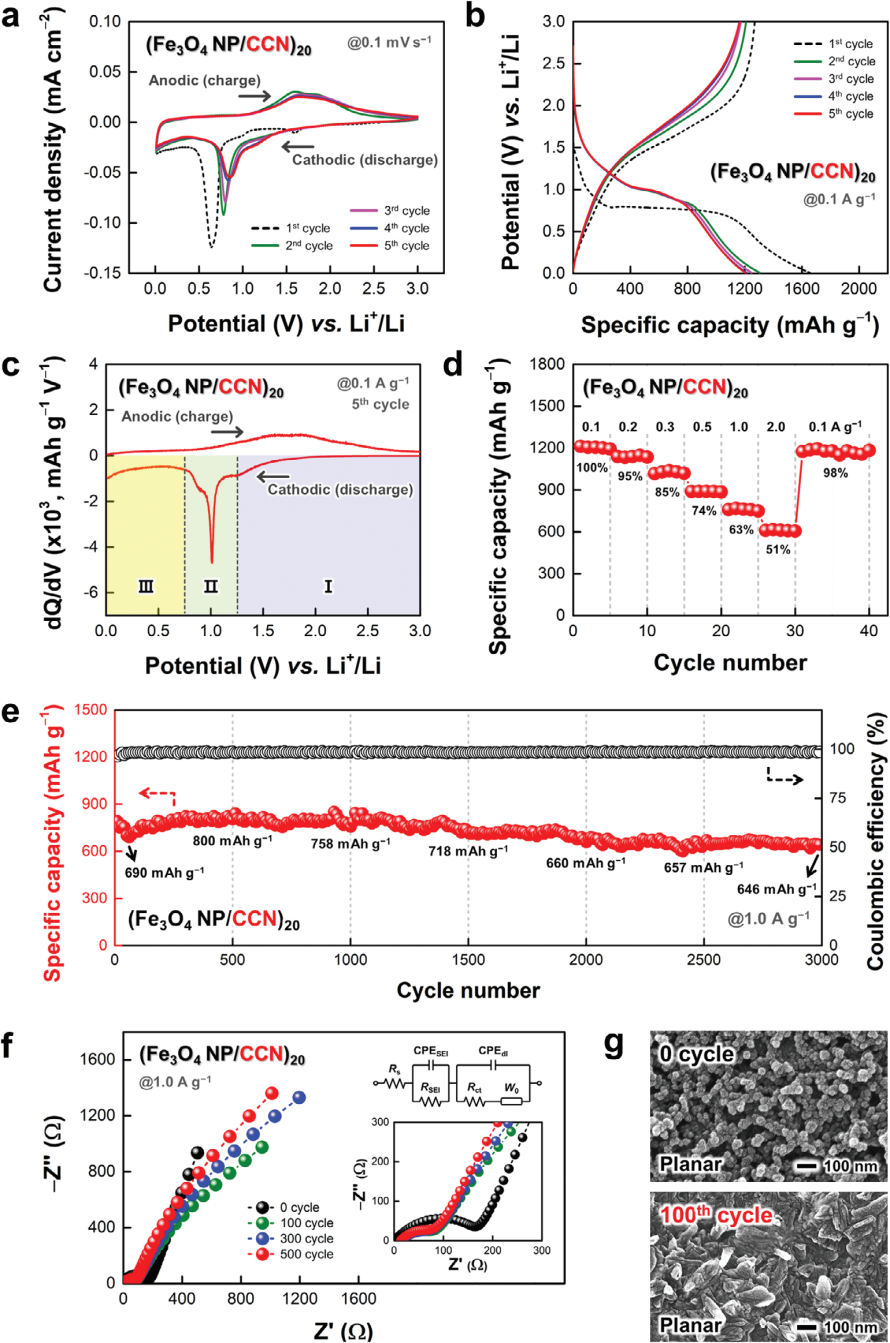
a) Cyclic voltammetry (CV) scans (at a scan rate of 0.1 mV s^−1^) and b) galvanostatic charge/discharge (GCD) profiles (at a current density of 0.1 A g^−1^) of (Fe_3_O_4_ NP/CCN)_20_‐LIB electrodes for the initial five cycles. c) Differential capacity plots of (Fe_3_O_4_ NP/CCN)_20_‐LIB electrodes at the 5^th^ cycle of GCD tests at a current density of 0.1 A g^−1^. d) Rate capabilities of (Fe_3_O_4_ NP/CCN)_20_‐LIB electrodes at different current densities ranging from 0.1 to 2.0 A g^−1^ after five initial activation cycles (at 0.1 A g^−1^). e) Long‐term operational stabilities of (Fe_3_O_4_ NP/CCN)_20_‐LIB electrodes during 3000 GCD cycles at a current density of 1.0 A g^−1^. f) Nyquist plots with an equivalent circuit (inset) of (Fe_3_O_4_ NP/CCN)_20_‐LIB electrodes at selected cycles (0, 100, 300, and 500 cycles) during GCD tests at a current density of 1.0 A g^−1^. g) Planar field‐emission scanning electron microscopy (FE–SEM) images of (Fe_3_O_4_ NP/CCN)_20_‐LIB electrodes before and after 100 GCD cycles at a current density of 1.0 A g^−1^. In this case, the specific capacities are calculated based on the total mass loading of the composites (≈0.11 mg cm^−2^), including Fe_3_O_4_ NPs and CCNs.

Based on the successful build‐up of CCN‐mediated Fe_3_O_4_ NP electrodes, we investigated the electrochemical properties to evaluate their applicability as LIB anodes. To this end, coin‐type half‐cells were prepared by assembling the (Fe_3_O_4_ NP/CCN)_20_ composites on flat Ni plates (denoted as (Fe_3_O_4_ NP/CCN)_20_‐LIB electrodes) and Li foils as counter/reference electrodes with a 1.0 m LiPF_6_ electrolyte. First, the Li storage behaviors of the electrodes were analyzed by cyclic voltammetry (CV) scans in the potential range of 0.01 to 3.0 V (vs. Li^+^/Li) at a scan rate of 0.1 mV s^−1^ (**Figure** **2**a). In the 1^st^ cathodic sweep, weak reduction peaks at ≈1.60 and 1.01 V were ascribed to the irreversible Li^+^ insertion into crystalline Fe_3_O_4_ to form Li*_x_* (Fe_3_O_4_) [Fe_3_O_4_ + *x*Li^+^ + *x*e^−^ → Li*_x_* (Fe_3_O_4_), up to *x* = 2]. Subsequently, a strong reduction peak at ≈0.64 V corresponded to the conversion reaction from Li*_x_* (Fe_3_O_4_) to Fe^0^/Li_2_O mixture [Li*_x_* (Fe_3_O_4_) + (8−*x*)Li^+^ + (8−*x*)e^−^ → 3Fe^0^ + 4Li_2_O] as well as the formation of an inorganic solid electrolyte interphase (SEI) layer caused by irreversible electrolyte decomposition. During the sequential anodic sweep, two broad oxidation peaks related to the reconversion reaction from Fe^0^ to Fe^2+^ and Fe^3+^ to reform Fe_3_O_4_ were identified at ≈1.60 and 1.86 V, respectively.^[^ ^42^, ^43^ ^]^ From the 2^nd^ CV cycle, both cathodic and anodic peaks slightly moved toward increased potential values, which indicated the enhanced redox kinetics due to the reduced polarization by the formation of nanograined Fe_3_O_4_ with smaller sizes than the original NPs during activation processes.^[^ ^44^ ^]^ Then, the subsequent CV curves gradually overlapped well with each other, suggesting the reversible and stable Li storage behaviors of the (Fe_3_O_4_ NP/CCN)_20_‐LIB electrodes.

Galvanostatic charge/discharge (GCD) measurements at a current density of 0.1 A g^−1^ also displayed the conversion reaction‐based redox behaviors of the (Fe_3_O_4_ NP/CCN)_20_‐LIB electrodes in good agreement with the CV results (Figure 2b). When the specific capacities were calculated based on the total mass loading of the composites (≈0.11 mg cm^−2^), the 1^st^ discharge cycle yielded a specific capacity of ≈1655 mAh g^−1^ with a relatively low Coulombic efficiency of ≈76.7%. This irreversible capacity loss in the initial cycle arose from both the unavoidable formation of an inorganic SEI layer and electrolyte decomposition, as commonly observed in nanostructured metal oxide‐based LIB anodes with a large active surface area.^[^ ^42^, ^43^, ^44^, ^45^, ^46^, ^47^, ^48^, ^49^ ^]^ At the 5^th^ cycle, the discharge capacity decreased to ≈1205 mAh g^−1^ with a high Coulombic efficiency of ≈96.5%; then, the capacity values remained almost constant with further cycling. Interestingly, the obtained capacity values far exceeded the theoretical capacity of Fe_3_O_4_ (≈924 mAh g^−1^). This phenomenon could be explained by (1) the partial contribution of CCNs and (2) the extra storage capacity of an electrolyte‐derived surface layer in the low potential range.^[^ ^50^, ^51^, ^52^ ^]^ Specifically, the CCN surface had numerous oxygen‐containing groups (i.e., C=O) that acted as redox sites, which could reversibly react with Li ions [Li^+^ + C=O + e^−^ ↔ C−O−Li] (Figure S11, Supporting Information). This result was consistent with previous reports on oxygen‐functionalized carbon materials in Li cells.^[^ ^53^, ^54^ ^]^ Thus, the CCNs exhibited a substantially higher specific capacity of ≈420 mAh g^−1^ compared to nonfunctionalized CNs (≈197 mAh g^−1^) with a double‐layer capacitance‐based Li storage mechanism. Additionally, to better understand the electrolyte‐derived surface layer, the differential capacity vs. potential profiles of the electrodes were plotted from the 5^th^ GCD cycle at a current density of 0.1 A g^−1^ (Figure 2c). In this case, the potential range was divided into three regions associated with (I) Li^+^ insertion into the Fe_3_O_4_ lattice (3.00−1.25 V), (II) the conversion reaction of nanograined Fe_3_O_4_ (1.25−0.75 V), and (III) the electrolyte‐derived surface layer (0.75−0.01 V). As confirmed in the potential range marked by region III, the capacity from the electrolyte‐derived surface layer accounted for a significant portion of the total capacity; this phenomenon evidently supported the existence of additional capacity in the low potential range. Although the origin of the additional capacity is not fully understood, the reversible formation of a polymeric gel‐like layer on the electrode surface from electrolyte decomposition has been suggested as a possible explanation.^[^ ^52^, ^55^ ^]^ To support this possibility, we monitored the morphology of the (Fe_3_O_4_ NP/CCN)_20_‐LIB electrodes in the lithiation state (after a discharge cycle) using HR–TEM. In this case, we could observe that many Fe_3_O_4_ nanograins with smaller sizes than the original NPs were surrounded by an amorphous matrix (Figure S12, Supporting Information). This result indicated the formation of the polymeric gel‐like layer due to the partial electrolyte decomposition at the interfaces between the electrode (Fe_3_O_4_ NPs) and the electrolyte.

To further understand the electrochemical behaviors of the (Fe_3_O_4_ NP/CCN)_20_‐LIB electrodes, we conducted scan rate‐dependent CV tests in the range of 0.1 to 1.0 mV s^−1^ (Figure S13a, Supporting Information). According to the power law [*i = av^b^*] between current density (*i*) and scan rate (*v*), the *b*‐values were obtained from the slopes in the plots of log *i* vs. log *v*. As shown in Figure S13b,c (Supporting Information), the electrodes exhibited the *b*‐values ranging from ≈0.40 to 0.80 under successive cathodic and anodic sweeps, indicating that their charge storage was induced by both surface‐ and diffusion‐controlled processes. In particular, the *b*‐value in the cathodic sweep of 0.75−0.01 V (region III in Figure 2c) showed a trend closer to 1.0, unlike other regions closer to 0.5 (including the conversion reaction in the cathodic sweep of 1.25−0.75 V (region II) and the Li ion insertion in the cathodic sweep of 2.00−1.25 V (region I) in Figure 2c), suggesting that the charge storage in the low potential range partially resulted from the surface‐controlled process caused by the electrolyte‐derived surface layer at the interfaces among Fe_3_O_4_ nanograins.^[^ ^56^ ^]^ Furthermore, the contribution ratios of surface‐ (*k*_1_ *v*) and diffusion‐controlled (*k*_2_ *v* ^1/2^) behaviors were quantitatively estimated using the equation [*i* = *k*_1_ *v* + *k*_2_ *v* ^1/2^], where *k*_1_ and *k*_2_ are constants at given potentials (Figure S13d,e, Supporting Information). As the scan rate increased from 0.1 to 1.0 mV s^−1^, the contribution of the diffusion‐controlled capacity gradually decreased from ≈83.3% to 60.4% due to the charge (ion) diffusion limitation at higher sweep rates, whereas the surface‐controlled capacity became more dominant. These results clearly demonstrated that the capacity of the (Fe_3_O_4_ NP/CCN)_20_‐LIB electrodes was influenced by the interplay of the surface‐ and diffusion‐controlled mechanisms, which strongly depended on the applied scan rate.

Then, the rate capabilities of the (Fe_3_O_4_ NP/CCN)_20_‐LIB electrodes were evaluated by measuring the specific capacities with progressively increasing current densities after five initial activation cycles at 0.1 A g^−1^ (Figure 2d and Figure S14, Supporting Information). As the current density increased from 0.1 to 2.0 A g^−1^, the average specific capacity decreased from ≈1205 to 610 mAh g^−1^, showing a relatively high‐capacity retention of ≈50.7%. When the current density decreased back to 0.1 A g^−1^, the specific capacities recovered up to ≈98.0% of the initial capacity, implying the good reversibility of the electrodes. Additionally, the (Fe_3_O_4_ NP/CCN)_20_‐LIB electrodes retained ≈23.4% of the initial capacity at 0.1 A g^−1^ as the current density increased up to 10 A g^−1^ (Figure S15, Supporting Information). The current density‐dependent areal and volumetric capacities of the electrodes were also recorded in Figure S16 (Supporting Information). In this case, the maximum areal and volumetric capacities were estimated to be ≈128 µAh cm^−2^ and ≈3013 mAh cm^−3^, respectively, at a current density of 0.1 A g^−1^. These outstanding capacity values with high‐rate performance could be explained by the fact that homogeneously distributed CCN networks within densely packed Fe_3_O_4_ NP arrays provided well‐interconnected charge transfer pathways, which enabled efficient Li storage behaviors despite the high electrode density.

Since metal oxide‐based LIB anodes inevitably involve the massive volume expansion of active materials (about 100% for Fe_3_O_4_) during lithiation,^[^ ^24^ ^]^ long‐term operational stabilities should be considered to ensure successful application in LIBs. For the (Fe_3_O_4_ NP/CCN)_20_‐LIB electrodes, their cycling performance was investigated during 3000 GCD cycles at a current density of 1.0 A g^−1^ (Figure 2e; Figure S17, Supporting Information). The initial discharge capacity was measured as ≈792 mAh g^−1^, which decreased to ≈690 mAh g^−1^ after 60 cycles and then increased to ≈800 mAh g^−1^ over 500 cycles. This gradual increase in the capacity (i.e., negative fading) with cycling was possibly attributed to the pseudocapacitance‐type behaviors of the abovementioned polymeric gel‐like layer on the electrode surface and/or the activation process.^[^ ^55^, ^57^ ^]^ As a result, the electrodes maintained ≈81.6% of their initial capacity (≈646 mAh g^−1^) with a high Coulombic efficiency of ≈99.0% even after 3000 cycles. These exceptional operational stabilities implied the excellent structural integrity of the electrodes through robust interfacial interactions between CCNs and Fe_3_O_4_, providing an effective safeguard against the repetitive volume changes in active Fe_3_O_4_.

To further understand the Li storage behaviors of the (Fe_3_O_4_ NP/CCN)_20_‐LIB electrodes during cycling tests, electrochemical impedance spectroscopy (EIS) analyses were conducted in the frequency range of 10^5^ to 0.1 Hz at the initial, 100^th^, 300^th^, and 500^th^ cycles using an equivalent circuit consisting of series resistance (*R*_s_), charge transfer resistance (*R*_ct_), Warburg impedance (*W*_0_), double‐layer capacitance (CPE_dl_), the resistance of the SEI layer (*R*_SEI_), and the capacitance of the SEI layer (CPE_SEI_) (Figure 2f, Figure S18, Supporting Information). First, as the cycle number increased from 0 to 100, the *R*_ct_ (from the semicircles in the high‐frequency region) notably decreased from ≈161 to 67.7 Ω. After 500 cycles, the *R*_ct_ was almost fixed at ≈66.9 Ω. These phenomena were mainly caused by the enhanced redox kinetics due to the formation of a stable SEI layer and nanograined Fe_3_O_4_ during cycling,^[^ ^44^, ^58^ ^]^ as already confirmed by the CV results. However, the ion diffusion resistance (from the slopes of tails in the low‐frequency region) increased to some degree with increasing cycle number and then gradually decreased with further cycling, which could be understood by observing the structural morphologies of the electrodes after 100 cycles. The highly nanoporous structure of the pristine electrodes were deconstructed after the 100^th^ cycle, forming a flower‐like amorphous structure by the nanoscale Kirkendall effect between fast‐diffusing Fe_3_O_4_ nanograins and relatively slow‐diffusing Li_2_O matrix (Figure 2g).^[^ ^52^, ^59^ ^]^ Additionally, the subsequent decrease in the ion diffusion resistance originated from the facile electrochemical access of reactive ions to the nanograined Fe_3_O_4_ by gradual activation with further cycling.^[^ ^43^, ^58^ ^]^ Along with the morphology changes of the (Fe_3_O_4_ NP/CCN)_20_‐LIB electrodes, XRD and FTIR measurements were carried out to further analyze the changes in the physical and chemical properties of the electrodes after cycling. As shown in Figure S19 (Supporting Information), the characteristic diffraction peaks of Fe_3_O_4_ NPs in the electrodes disappeared after cycling, suggesting the transformation of crystalline Fe_3_O_4_ NPs into an amorphous phase. On the other hand, the newly emerged diffraction peak at ≈33.6° indicated the (111) plane of Li_2_O matrix in the formed SEI layer on the electrode surface. These results were consistent with previous studies on conversion‐type metal oxides for LIB anodes.^[^ ^24^, ^44^, ^59^ ^]^ Furthermore, the FTIR spectra of the electrodes confirmed the generation of the SEI layer after cycling through the presence of Li_2_CO_3_ at 1550−1350, 1086, and 866 cm^−1^, which is known as a major component of the SEI layer (Figure S20, Supporting Information).^[^ ^59^, ^60^, ^61^ ^]^

### 2.3 FCC‐Based LIB Electrodes Using a CCN‐Mediated Fe_3_O_4_ NP Assembly

Although thin film‐type (Fe_3_O_4_ NP/CCN)*_n_* composites could achieve attractive characteristics regarding specific/volumetric capacities, rate capabilities, and operational stabilities in LIBs, their low areal performance levels resulting from the low mass loading of active components limited their practical use. Although the mass loading of (Fe_3_O_4_ NP/CCN)*_n_* composites could be further increased by increasing bilayer number, the thickened electrode layers inevitably increased charge transfer length, adversely affecting the overall energy efficiency.^[^ ^28^ ^]^ To resolve this critical drawback, we employed 3D porous FCCs with a large specific surface area instead of nonporous flat current collectors. In this case, the porous FCCs were prepared through the carbonization‐assisted Ni electrodeposition of textiles (see the Experimental Section; Figure S21, Supporting Information). The prepared 650 µm‐thick FCCs exhibited a significantly low sheet resistance of ≈0.02 Ω sq^−1^, a mass density of ≈31.1 mg cm^−2^, and highly interconnected fibril structures with numerous voids for facile charge transport. Due to the structural benefits of porous FCCs, the mass loading of (Fe_3_O_4_ NP/CCN)_20_ composites on the FCCs (denoted as (Fe_3_O_4_ NP/CCN)_20_‐FCCs) increased to values more than ≈54 times higher than that on the flat current collectors (i.e., Ni plates) (Figure S22, Supporting Information). As a result, the total mass loading of the composites on the FCCs was measured to be ≈6.2 ± 0.1 mg cm^−2^ with the mass ratio of active Fe_3_O_4_ NPs estimated to be ≈85.5%.

**Figure 3:**
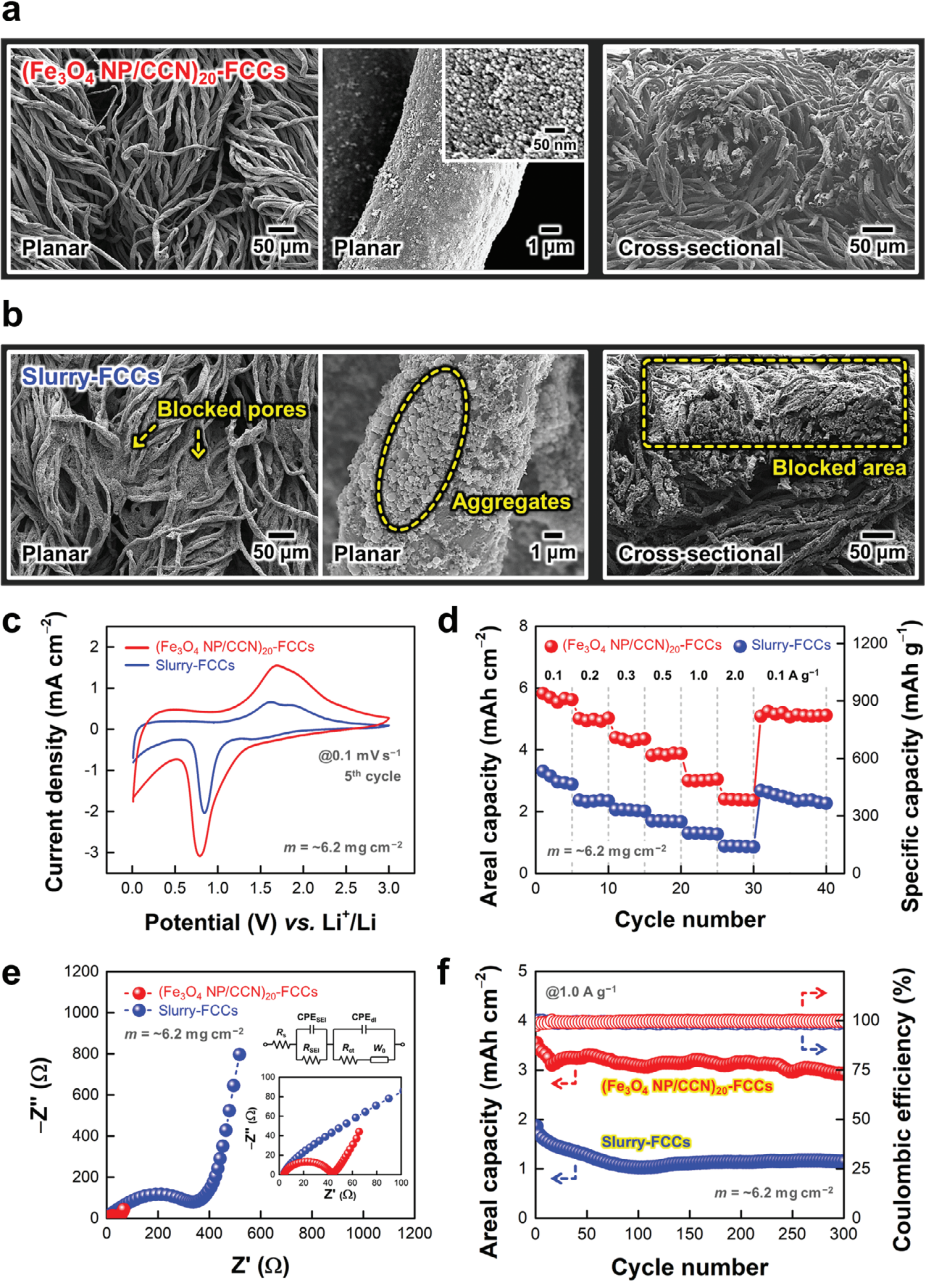
Low‐magnification planar (left), high‐magnification planar (middle), and cross‐sectional (right) field‐emission scanning electron microscopy (FE–SEM) images of a) (Fe_3_O_4_ NP/CCN)_20_‐FCCs and b) slurry‐FCCs. c) Comparison of cyclic voltammetry (CV) curves at a scan rate of 0.1 mV s^−1^ between (Fe_3_O_4_ NP/CCN)_20_‐FCCs and slurry‐FCCs at the 5^th^ cycle. d) Comparison of areal capacities (left axis) and specific capacities (right axis) between (Fe_3_O_4_ NP/CCN)_20_‐FCCs and slurry‐FCCs with varying current density from 0.1 to 2.0 A g^−1^ after five initial activation cycles (at 0.1 A g^−1^). In this case, the specific capacities are calculated based on the total mass loading of the composites (≈6.2 mg cm^−2^) on FCCs. e) Comparison of Nyquist plots between (Fe_3_O_4_ NP/CCN)_20_‐FCCs and slurry‐FCCs. The inset shows the equivalent circuit of the electrodes. f) Comparison of long‐term operational stabilities between (Fe_3_O_4_ NP/CCN)_20_‐FCCs and slurry‐FCCs during 300 galvanostatic charge/discharge (GCD) cycles at a current density of 1.0 A g^−1^. In this case, the total mass loading (*m*) of two FCC‐based electrodes is equally adjusted to ≈6.2 mg cm^−2^.

In particular, our CCN‐mediated nanoblending assembly effectively incorporated the electrode components into all accessible regions ranging from the exterior to the interior of porous FCCs. Despite the high total mass loading of the composites (≈6.2 mg cm^−2^), the Fe_3_O_4_ NP arrays were uniformly and densely assembled on the entire surface of the porous structure without any notable NP agglomeration and/or pore clogging, as confirmed by FE–SEM and EDS elemental mapping (**Figure** **3**a; Figure S23, Supporting Information). Additionally, we prepared comparative samples using mechanically blended slurries, composed of commercial Fe_3_O_4_ nanopowders, pristine CNs, and COOH‐functionalized polymeric binders (poly(acrylic acid); PAA). In this case, the slurry‐coated FCCs (denoted slurry‐FCCs) displayed nonuniform fibril structures with aggregated phases and blocked pores (Figure 3b), which occurred mainly due to the high viscosity of slurries and unfavorable interfacial interactions among electrode components. Particularly, since the aggregates of poorly conductive Fe_3_O_4_ NPs and the blocked pores could severely restrict the charge transfer within electrodes and the full utilization of the large surface area of the FCCs,^[^ ^27^ ^]^ it was reasonable to expect that the performance of slurry‐FCCs was inferior to that of the (Fe_3_O_4_ NP/CCN)_20_‐FCCs.

To confirm the effects of porous FCCs and uniformity of electrode layers on energy storage performance, the electrochemical properties of (Fe_3_O_4_ NP/CCN)_20_‐FCCs and slurry‐FCCs were investigated at the same total mass loading (≈6.2 mg cm^−2^). As shown in Figure 3c, the (Fe_3_O_4_ NP/CCN)_20_‐FCCs displayed higher current responses than the slurry‐FCCs in the 5^th^ CV scan at a scan rate of 0.1 mV s^−1^. In this case, the areal charge density from the integral CV area of the (Fe_3_O_4_ NP/CCN)_20_‐FCCs was ≈48 times higher than that of the flat substrate‐based (Fe_3_O_4_ NP/CCN)_20_‐LIB electrodes while maintaining a prominent redox pair of the conversion reaction of Fe_3_O_4_. These results evidently indicated that the porous FCCs enhanced the areal performance levels of the electrodes by allowing the high mass loading of Fe_3_O_4_ NPs without any notable loss of their charge transfer kinetics. Furthermore, the structural benefits of the (Fe_3_O_4_ NP/CCN)_20_‐FCCs with superior coating quality enabled more efficient charge transfer behaviors at electrode/electrolyte interfaces even at higher sweep rates compared to the slurry‐FCCs. The scan rate‐dependent CV measurements for each electrode revealed that the (Fe_3_O_4_ NP/CCN)_20_‐FCCs exhibited much higher charge densities with smaller redox peak separation (Δ*E*_p_) values (≈1.26 V at 1.0 mV s^−1^) compared to the slurry‐FCCs (≈1.38 V), suggesting the better charge transfer kinetics of CCN‐mediated Fe_3_O_4_ NP arrays (Figure S24, Supporting Information). In addition, a bare FCC also displayed Li storage behaviors due to the formation of native Ni oxides on the electrodeposited Ni layer (Figure S25, Supporting Information); however, the capacity of the (Fe_3_O_4_ NP/CCN)_20_‐FCCs was dominantly governed by the CCN‐mediated Fe_3_O_4_ NP arrays.

To further confirm the advantages of porous FCCs, we investigated the current density‐dependent GCD profiles of (Fe_3_O_4_ NP/CCN)_20_‐FCCs and slurry‐FCCs (Figure 3d; Figure S26, Supporting Information). In this case, all GCD measurements were recorded after five initial activation cycles at a current density of 0.1 A g^−1^. As a result, the (Fe_3_O_4_ NP/CCN)_20_‐FCCs delivered a maximum average areal capacity of ≈5.67 mAh cm^−2^ at 0.1 A g^−1^ and maintained ≈42.0% (≈2.38 mAh cm^−2^) of the initial capacity at 2.0 A g^−1^. However, the slurry‐FCCs displayed an output areal capacity of ≈3.04 mAh cm^−2^ at 0.1 A g^−1^ with a capacity retention of ≈28.6% (≈0.87 mAh cm^−2^) at 2.0 A g^−1^. Furthermore, as the current density returned from 2.0 to 0.1 A g^−1^, the average areal capacity of the slurry‐FCCs retained only ≈80.1% of their original value with a continuous capacity decay, which was in stark contrast to the (Fe_3_O_4_ NP/CCN)_20_‐FCCs with a capacity recovery of ≈90.5%. These rate performance trends of each electrode showed a more pronounced difference as the current density increased up to 10 A g^−1^ (Figure S27, Supporting Information), clearly indicating that the uniformity of electrode layers on current collectors (i.e., FCCs) was a critical determinant for achieving better rate performance and energy efficiency. A substantial difference in the performance levels between two different kinds of FCC‐based electrodes were further confirmed by EIS analyses (Figure 3e, Figure S28, Supporting Information). The (Fe_3_O_4_ NP/CCN)_20_‐FCCs showed a considerably lower *R*_ct_ value (≈44 Ω) than the slurry‐FCCs (≈346 Ω), implying superior charge transfer kinetics at electrode/electrolyte interfaces. Notably, the *R*_ct_ of the (Fe_3_O_4_ NP/CCN)_20_‐FCCs was even smaller than that of the flat substrate‐based (Fe_3_O_4_ NP/CCN)_20_‐LIB electrodes (*R*_ct_ =161 Ω; Figure 2f) despite the much higher mass loading of Fe_3_O_4_ NPs. Additionally, the (Fe_3_O_4_ NP/CCN)_20_‐FCCs retained ≈82.5% (≈2.93 mAh cm^−2^) of their initial capacity with a high Coulombic efficiency of ≈99.7% after 300 GCD cycles at a current density of 1.0 A g^−1^, while the slurry‐FCCs yielded a capacity retention of ≈61.3% (≈1.14 mAh cm^−2^) (Figure 3f). As a result, our direct CCN‐mediated nanoblending assembly was highly beneficial for deriving the spatial distribution/arrangement of electrode components and exploiting the large surface area of porous FCCs.

**Figure 4:**
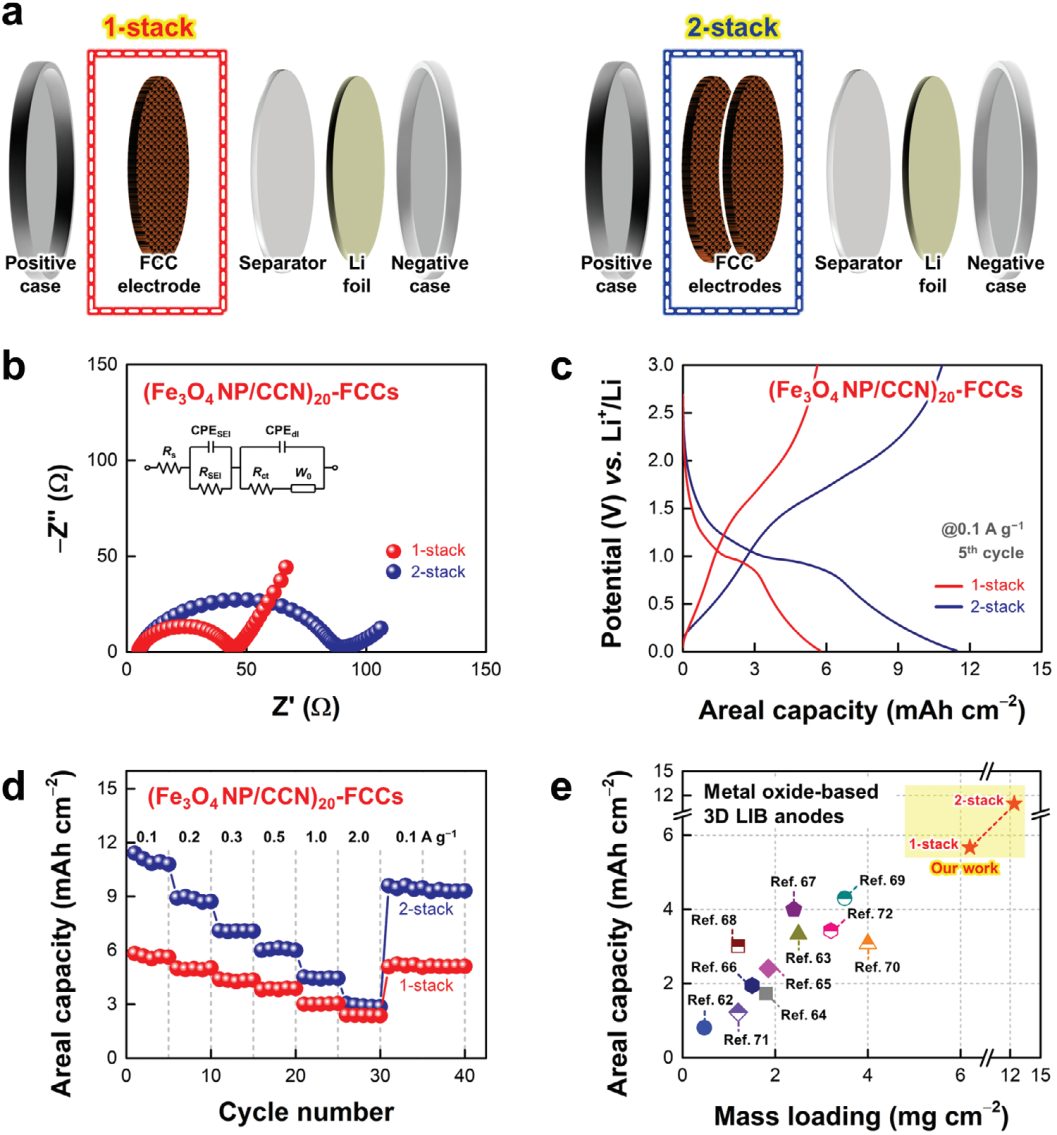
a) Schematic diagrams for the multistacking of (Fe_3_O_4_ NP/CCN)_20_‐FCCs. b) Nyquist plots and c) galvanostatic charge/discharge (GCD) profiles (5^th^ cycle, at a current density of 0.1 A g^−1^) of (Fe_3_O_4_ NP/CCN)_20_‐FCCs as a function of stacking number. d) Rate capabilities of (Fe_3_O_4_ NP/CCN)_20_‐FCCs with varying current density from 0.1 to 2.0 A g^−1^ after five initial activation cycles (at 0.1 A g^−1^) as a function of stacking number. e) Comparison of areal capacities of (Fe_3_O_4_ NP/CCN)_20_‐FCCs with metal oxide‐based 3D lithium‐ion battery (LIB) anodes using porous current collectors in the literatures.

Another notable benefit of using porous FCCs is that the areal performance levels can be further enhanced through the simple multistacking of electrodes (**Figure** **4**a). Although the overall thickness of multistacked electrodes increased, the porous fibril structures of FCCs allowed facile charge transport to the inner regions of the multistacked electrodes. Specifically, the *R*_ct_ of 2‐stacked (Fe_3_O_4_ NP/CCN)_20_‐FCCs increased from ≈44 to 91 Ω due to the extended charge transfer length, along with the increased ion diffusion resistance, as confirmed by EIS analyses (Figure 4b). However, the 2‐stacked electrodes yielded a significantly increased areal capacity of ≈11.0 mAh cm^−2^ at a current density of 0.1 A g^−1^, which was almost proportional to the increase in the total mass loading of the electrodes (≈12.4 mg cm^−2^ for 2‐stack) (Figure 4c). Regarding specific capacities and rate capabilities, the 2‐stacked electrodes showed relatively lower specific capacities over the range of current densities and a capacity retention of ≈26.6% (≈2.93 mAh cm^−2^) at 2.0 A g^−1^, compared to the 1‐stacked electrodes with a capacity retention of ≈42.0%, which was mainly caused by the increased total resistance (Figure 4d; Figure S29, Supporting Information). Particularly, the areal capacities of our (Fe_3_O_4_ NP/CCN)_20_‐FCCs outperformed those of previously reported metal oxide‐based 3D LIB anodes using porous current collectors (Figure 4e; Table S1, Supporting Information).^[^ ^62^, ^63^, ^64^, ^65^, ^66^, ^67^, ^68^, ^69^, ^70^, ^71^, ^72^ ^]^ That is, since a substantial increase in the mass loading of active materials inevitably involves performance degradation, our approach, which can induce the effective spatial distribution/arrangement of components on porous (or multistacked) FCCs, is highly efficient for understanding interfacial interaction/structure‐dependent charge transfer and further achieving high‐performance energy storage electrodes.

## 3 Conclusion

In summary, we introduced a unique CCN‐mediated nanoblending assembly for preparing binder‐free energy storage electrodes. In particular, our approach was characterized by the fact that the effective spatial distribution/arrangement of electrode components could be realized by favorable interfacial interactions between high‐energy MO NPs and conductive CCNs. That is, the COOH groups of CCNs had high interfacial affinities for the surface of MO NPs; these affinities strongly induced an in situ ligand‐exchange reaction between bulky/insulating ligands and CCNs on the NP surface through multidentate binding during the consecutive LbL assembly. Thus, the CCNs acted as robust linkers for directly bridging all interfaces between neighboring MO NPs to form well‐interconnected charge transfer pathways. Based on this interfacial design, the CCN‐mediated Fe_3_O_4_ NP electrodes exhibited high capacities, good rate capabilities, and long‐term operational stabilities as LIB anodes. Additionally, when our approach was applied to highly porous FCCs, the areal capacities were markedly enhanced to ≈5.67 mAh cm^−2^ (1‐stack) and ≈11.0 mAh cm^−2^ (2‐stack) by fully exploiting the large surface area of FCCs. Our approach provides a basis for understanding the importance of interfacial interaction/structure‐dependent charge transfer processes and further developing high‐performance energy storage electrodes.

## 4 Experimental Section

The detailed experimental information are available in the part of the Supporting Information.

## Conflict of Interest

The authors declare no conflict of interest.

## Supporting information

Supporting Information

## Data Availability

The data that support the findings of this study are available from the corresponding author upon reasonable request.
