# Supplementary material for: Carbon Nanocluster‐Mediated Nanoblending Assembly for Binder‐Free Energy Storage Electrodes with High Capacities and Enhanced Charge Transfer Kinetics
Source: Adv Sci (Weinh). 2023 May 21;10(22):2301248. doi: 10.1002/advs.202301248 (PMC10401157; doi:10.1002/advs.202301248)
Supplement: Supplementary file 1 — Supporting Information [file ADVS-10-2301248-s001.pdf]

ADVANCED SCIENCE Open Access

Supporting Information

for *Adv. Sci.*, DOI 10.1002/advs.202301248

Carbon Nanocluster-Mediated Nanoblending Assembly for Binder-Free Energy Storage Electrodes with High Capacities and Enhanced Charge Transfer Kinetics

Yongkwon Song, Woojin Bae, Jeongyeon Ahn, Youhyun Son, Minseong Kwon, Cheong Hoon Kwon, Younghoon Kim, Yongmin Ko and Jinhan Cho**

Supporting Information

Carbon Nanocluster-Mediated Nanoblending Assembly for Binder-Free Energy Storage Electrodes with High Capacities and Enhanced Charge Transfer Kinetics

Yongkwon Song,[†] Woojin Bae,[†] Jeongyeon Ahn, Youhyun Son, Minseong Kwon, Cheong Hoon Kwon, Younghoon Kim, Yongmin Ko, and Jinhan Cho**

[‡] These authors contributed equally to this work.

Experimental Section

Materials: Iron(III) acetylacetonate (Fe(acac)$_3$; 97%), 1,2-hexadecanediol (90%), oleic acid (OA; 90%), oleylamine (OAm; 70%), benzyl ether (98%), nickel(II) chloride hexahydrate ($NiCl_2 \cdot 6H_2O$; *ReagentPlus*®), nickel(II) sulfate hexahydrate ($NiSO_4 \cdot 6H_2O$; ≥98%), boric acid (H_3BO_3; ≥99.5%), polyethyleneimine (PEI; branched, M_w ~800), poly(acrylic acid) (PAA; M_v ~450,000), Fe_3O_4 nanopowders (particle size: 50−100 nm, 97%), and fluoroethylene carbonate (FEC; 99%) were supplied by Sigma–Aldrich. Organic solvents (ethanol and toluene), sulfuric acid (H_2SO_4; 98%), and nitric acid (HNO_3; 60%) were obtained from Daejung Chemicals & Metals (Republic of Korea). Carbon nanoclusters (CNs; EQ-Lib-Super P) were purchased from MTI Korea (Republic of Korea). An organic electrolyte of lithium hexafluorophosphate ($LiPF_6$; 1.0 mol L^{-1}) in a mixture of ethylene carbonate (EC) and dimethyl carbonate (DMC) with a volume ratio of 3:7 was supplied by Dongwha Electrolyte (Republic of Korea). All chemical reagents were used as received without further purification.

Synthesis of OA-Fe_3O_4 NPs: Monodisperse OA-Fe_3O_4 NPs with an average diameter of ~7 nm in toluene were prepared using a previously reported protocol.[S1] A mixture of Fe(acac)$_3$ (2 mmol), 1,2-hexadecanediol (10 mmol), OA (6 mmol), OAm (6 mmol), and benzyl ether (20 mL) was added to a three-neck flask with vigorous stirring under a flow of inert gas. Next, the reaction mixture was heated at 200 °C for 2 h and sequentially heated at 300 °C to reflux for 1 h. After cooling the flask to room temperature by removing a heating mantle, excess ethanol was added to precipitate OA-Fe_3O_4 NPs, followed by centrifugation (8,000 rpm, 10 min). The separated OA-Fe_3O_4 NPs were dissolved in toluene containing OA (0.05 mL) and OAm (0.05 mL). Additional centrifugation (8,000 rpm, 10 min) with excess ethanol was carried out several times to collect pure OA-Fe_3O_4 NPs, which were redissolved in toluene for further use.

Synthesis of CCNs: COOH-functionalized CNs (CCNs) were prepared through an acid treatment of hydrophobic CNs. Briefly, CNs (0.5 g) were surface-treated in a mixture of H_2SO_4

(30 mL) and HNO_3 (10 mL) at 70 °C for 3 h in a one-neck flask with magnetic stirring under an atmospheric condition. After the reaction was completed and cooled to room temperature, the mixture was slowly purified using deionized water, followed by centrifugation (10000 rpm, 10 min) and vacuum filtration to fully eliminate residual acid. The vacuum-filtrated CCN films were completely dried in a vacuum oven and dissolved in ethanol for further use.

Preparation of FCCs: The porous FCCs were prepared by the carbonization-assisted Ni electrodeposition of cotton textiles. First, the bare textiles were washed with deionized water and dried in a vacuum oven at room temperature. Then, the cleaned textiles were carbonized by heating to 950 °C at a rate of 3 °C min^{-1} and maintaining for 3 h in a tube furnace under a flow of N_2 gas. After cooling to room temperature, the carbonized textiles were Ni-electrodeposited in a Watt bath (45 g L^{-1} of $NiCl_2$, 240 g L^{-1} of $NiSO_4$, and 30 g L^{-1} of H_3BO_3) based on a two-electrode system with a high-purity Ni plate as a counter electrode. The Ni electrodeposition was conducted at a current density of 100 mA cm^{-2} for 20 min using a power supply, followed by washing with deionized water and drying in a vacuum oven at room temperature.

LbL Assembly of (Fe_3O_4 NP/CCN)$_n$ Composites: First, all experimental procedures for LbL assembly were conducted in a fume hood for safety. The solution concentrations of OA-Fe_3O_4 NPs (in toluene) and CCNs (in ethanol) were adjusted to 10 and 2 mg mL^{-1}, respectively. Flat substrates (including Si wafers, SiO_2/Si wafers, gold-sputtered Si wafers, quartz glasses, QCM electrodes, FTO glasses, and Ni plates) were surface-treated using a UV–ozone cleaner for 30 min, whereas porous FCCs were used without additional treatment. The surface-treated substrates were immersed in an amine (NH_2)-functionalized PEI solution (2 mg mL^{-1} in ethanol) for 30 min to generate a robust underlayer, followed by washing with pure ethanol to remove weakly adsorbed materials and drying to remove residual solvent. After that, the PEI-coated substrates were immersed in an OA-Fe_3O_4 NP solution (followed by washing with toluene and drying) and a CCN solution (followed by washing with ethanol and drying) for 10 min for each

component, producing one bilayer of (Fe_3O_4 NP/linker)$_n$ composites. These procedures were repeated until the desired bilayer number (n) of composites was obtained.

Preparation of Slurry-FCCs: Slurries were prepared by the mechanical dispersion of active Fe_3O_4 nanopowders, conductive CNs, and COOH-functionalized PAA binder with a weight ratio of 8:1:1 in ethanol. Then, the slurries were deposited on porous FCCs using a dip-coating method, followed by drying at 120 °C in a vacuum oven for 8 h to obtain slurry-FCCs.

Characterization: The size/shape and crystal structures of OA-Fe_3O_4 NPs and CCNs were investigated by HR–TEM (Tecnai F20, FEI) and XRD (SmartLab, Rigaku) with Cu K_α radiation (45 kV, 200 mA). FTIR measurements of pristine materials and composites on gold-sputtered Si wafers were conducted using a Cary 600 (Agilent Technologies) in an attenuated total reflection (ATR) mode at a resolution of 2 cm^{-1}. The obtained FTIR spectra were plotted after smoothing and baseline correction using a spectral analysis software (OMNIC, Thermo Fisher Scientific). A contact angle apparatus (Phoenix-300, SEO Corp.) was used to measure the water contact angles of composites on Si wafers using deionized water with a pH of ~5.8. The qualitative growth of composites on quartz glasses was monitored by UV–vis spectroscopy (Lambda 365, Perkin Elmer) in the scan range of 800 to 200 nm. The mass changes (Δm, μg cm^{-2}) of composites on QCM electrodes were calculated from the frequency changes ($-\Delta F$, Hz) acquired using a QCM 200 (SRS) according to the Sauerbrey Equation (1) as follows.[S2]

$$\Delta F\ (\mathrm{Hz}) = -\frac{2F_0^2}{A\sqrt{\rho_\mathrm{q}\mu_\mathrm{q}}} \times \Delta m \tag{1}$$

In Equation (1), F_0 is the fundamental resonance frequency (~5 MHz), A is the surface area (cm^2), ρ_q is the density (~2.65 g cm^{-3}), and μ_q is the shear modulus (~2.95 × 10^{11} g cm^{-1} s^{-2}) of the QCM electrodes. Thus, Equation (1) was expressed as Equation (2) by substituting actual values into the variables.

$$-\Delta F = 56.6 \times \Delta m \tag{2}$$

The morphologies and elemental mapping of composites on Si wafers were characterized by FE–SEM (S-4800, Hitachi) equipped with EDS. A semiconductor parametric analyzer (Agilent 4155B, Agilent Technologies) was used to measure the electrical conductivity of spin-coated CCN films on SiO_2/Si wafers through a four-probe method and to record the current–voltage profiles of composites on gold-sputtered Si wafers through a two-probe method using gold wires (with a diameter of ~0.5 mm) as top/bottom electrodes.

Electrochemical Measurements: To evaluate the electrochemical performance of LIB electrodes (using Ni plates or porous FCCs as current collectors), half-cells were assembled in an argon-filled glove box (MBraun, O_2 <0.1 ppm, H_2O <0.1 ppm) using CR2032-type coin cells with Li metal foils (as counter/reference electrodes) and Celgard separators. A solution of $LiPF_6$ (1.0 mol L^{-1}) dissolved in a mixture of EC/DMC (3:7, *v*/*v*) with an additive of FEC (10 wt%) was used as an organic electrolyte. A battery cycler (WBCS3000, WonATech) was employed to investigate the assembled coin cells in the potential range of 0.01 to 3.0 V (*vs.* Li^+/Li). EIS analyses were conducted in the frequency range of 10^5 to 0.01 Hz with an amplitude of 0.01 mV using an impedance analyzer (ZIVE MP2, WonATech). The specific capacities of the LIB electrodes were calculated based on the total mass loading of the composites, including Fe_3O_4 NPs and CCNs, on current collectors. In this case, the mass loading was measured using a QCM for flat current collector-based electrodes and an analytical balance (ABT 220-5DNM, KERN & SOHN GmbH) with a readability of 0.1 mg for FCC-based electrodes. The separation of surface- (k_1v) and diffusion-controlled ($k_2v^{1/2}$) contribution in scan rate-dependent CV curves was carried out using the Equation (3) between current (i) and scan rate (v):[S3]

$$i(V) = k_1v + k_2v^{1/2} \tag{3}$$

where k_1 and k_2 are constants at given potentials (V). That is, the values for k_1 and k_2 can be obtained by plotting $i(V)/v^{1/2}$ *vs.* $v^{1/2}$, where k_1 and k_2 are the slopes and y-intercepts, respectively.

a

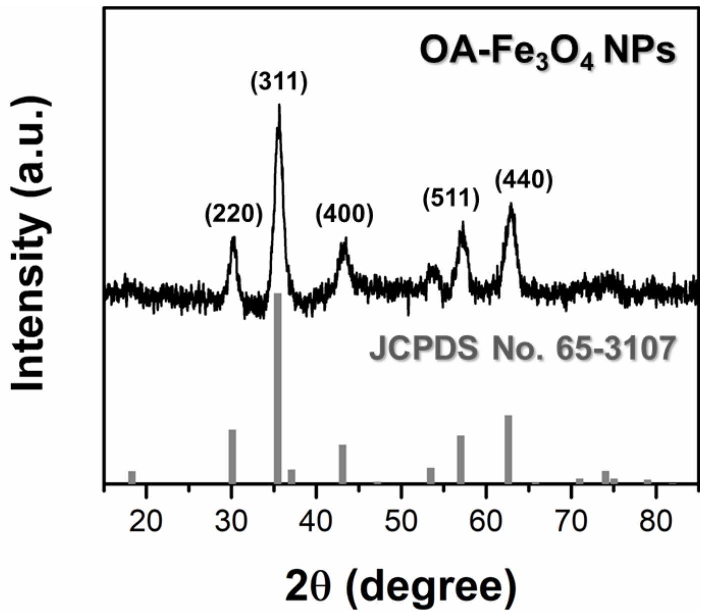

b

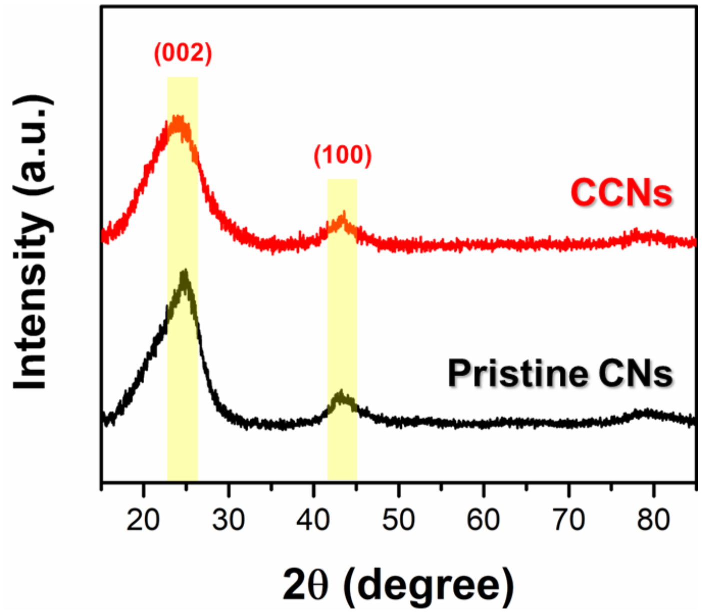

Figure S1. a) XRD patterns of synthesized OA-Fe_3O_4 NPs (top) with referential cubic spinel-structured Fe_3O_4 crystals (bottom; JCPDS card No. 65-3107).[S4] b) XRD patterns of interface-modified CCNs (top) and pristine CNs (bottom).

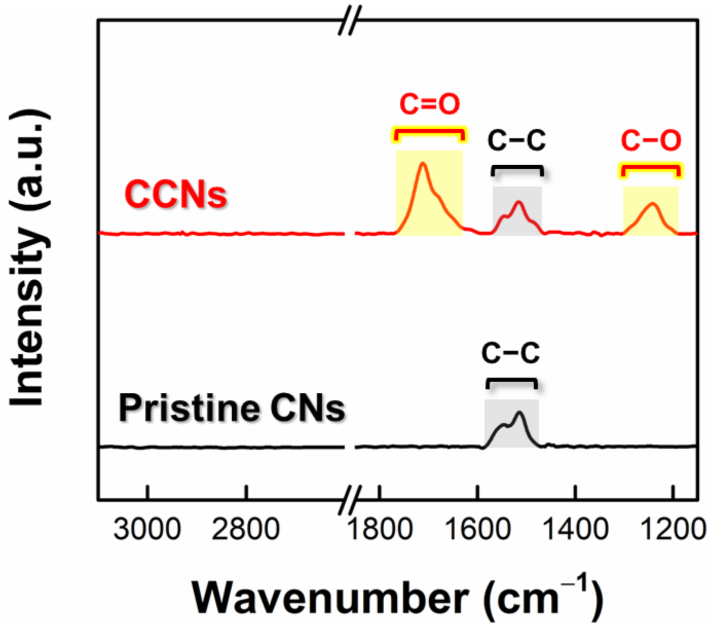

Figure S2. FTIR spectra of interface-modified CCNs (top) and pristine CNs (bottom).

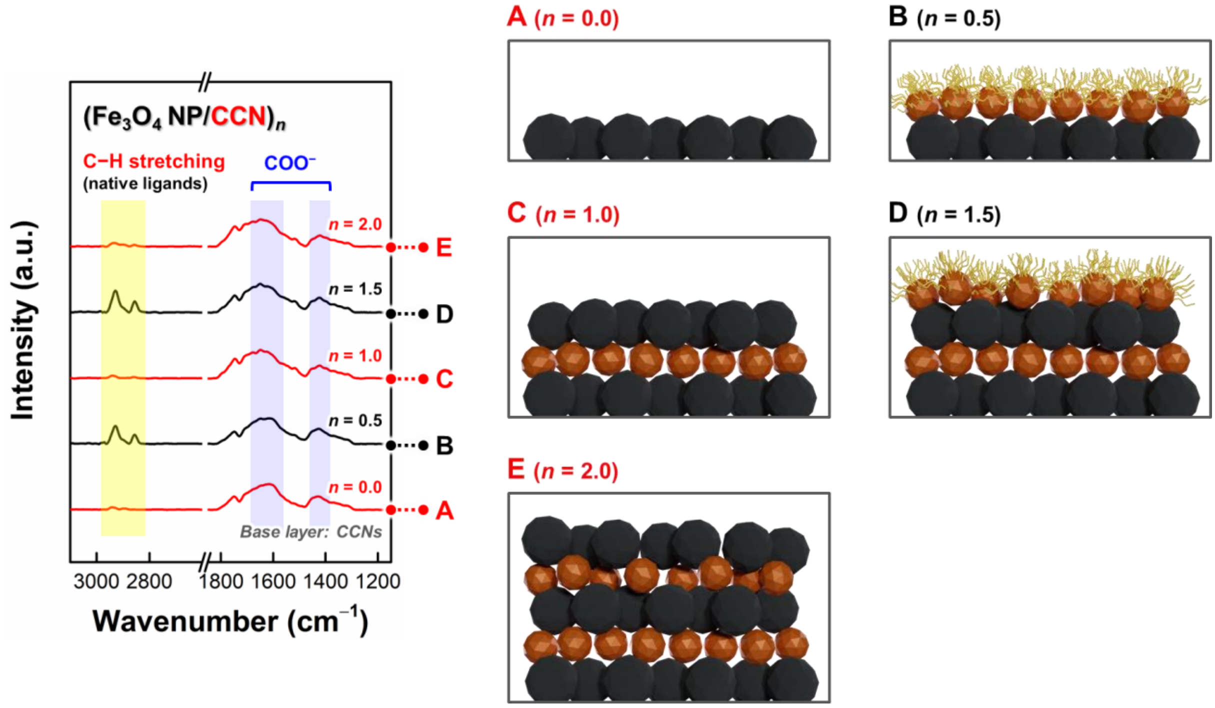

Figure S3. Bilayer-dependent FTIR spectra and the corresponding schematic representations of (Fe_3O_4 NP/CCN)$_n$ composites. The periodic changes in the peak intensity for C−H stretching vibrations (at 3000−2800 cm^{-2}) indicated that the native OA/OAm ligands bound to the surface of Fe_3O_4 NPs were successfully removed and replaced by CCNs, as illustrated in the schemes. While we presented a highly ordered interface between Fe_3O_4 NPs and CCNs in the schemes, this description was somewhat exaggerated to aid in understanding the ligand exchange reaction. In reality, the structure of the (Fe_3O_4 NP/CCN)$_n$ composites was a well-nanoblended layered structure.

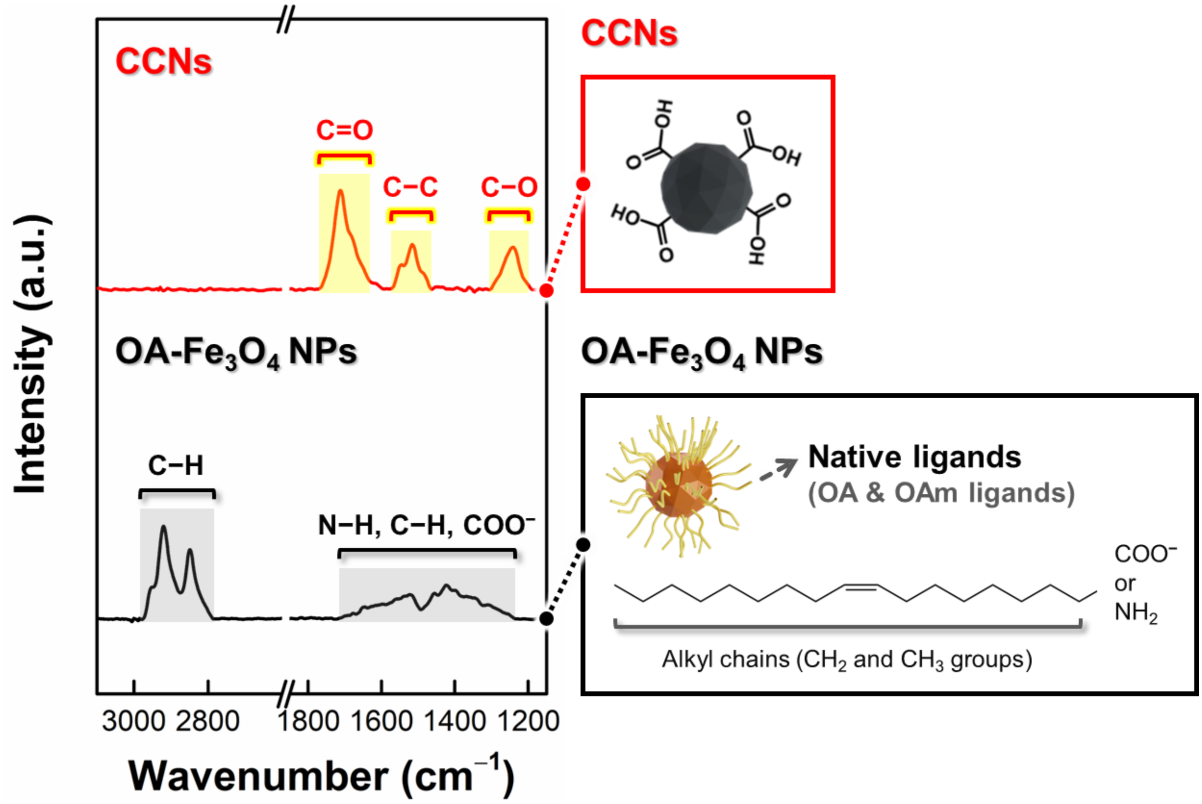

Figure S4. FTIR spectra and schematic illustrations of a) interface-modified CCNs (top) and b) OA-Fe_3O_4 NPs (bottom). In this case, the OA-Fe_3O_4 NPs showed distinct C−H stretching peaks in the range of 3000 to 2800 cm^{-1} and the overlapped absorption band (by N−H bending, C−H bending, and COO^- stretching) in the range of 1700 to 1200 cm^{-1}, which were assigned to the native OA/OAm ligands on the surface of Fe_3O_4 NPs.[S5]

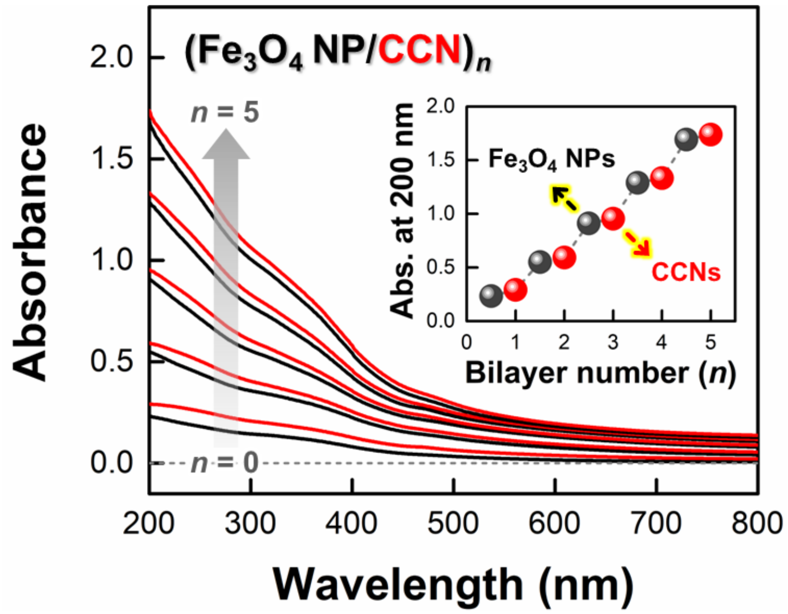

Figure S5. UV–vis absorbance spectra and the values at the wavelength of 200 nm (inset) of (Fe_3O_4 NP/CCN)$_n$ composites as the bilayer number (n) increased 0 to 5. The black line/circles and red line/circles indicate the deposition of OA-Fe_3O_4 NPs and CCNs, respectively.

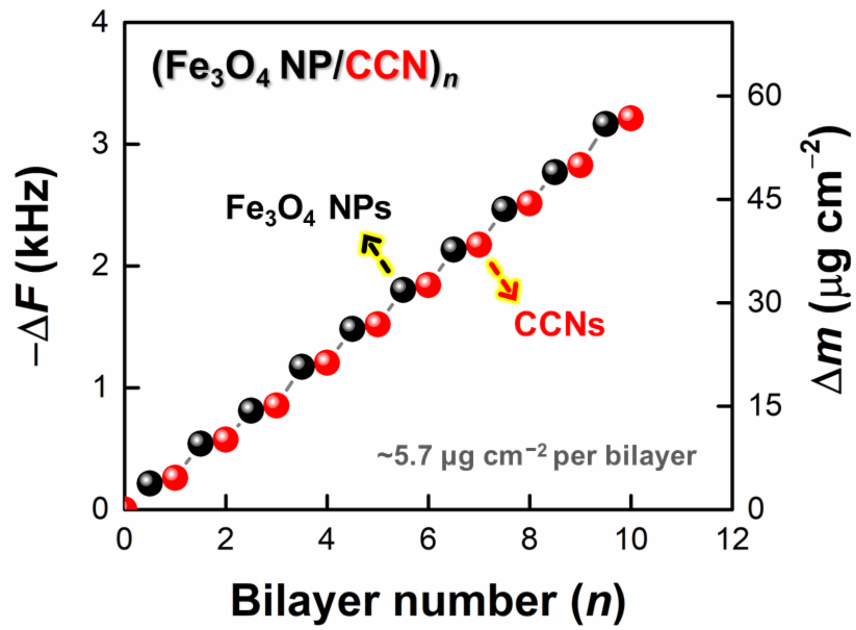

Figure S6. Frequency ($-\Delta F$, left axis) and mass (Δm, right axis) changes of (Fe_3O_4 NP/CCN)$_n$ composites obtained by using QCM with increasing bilayer number (n) from 0 to 10.

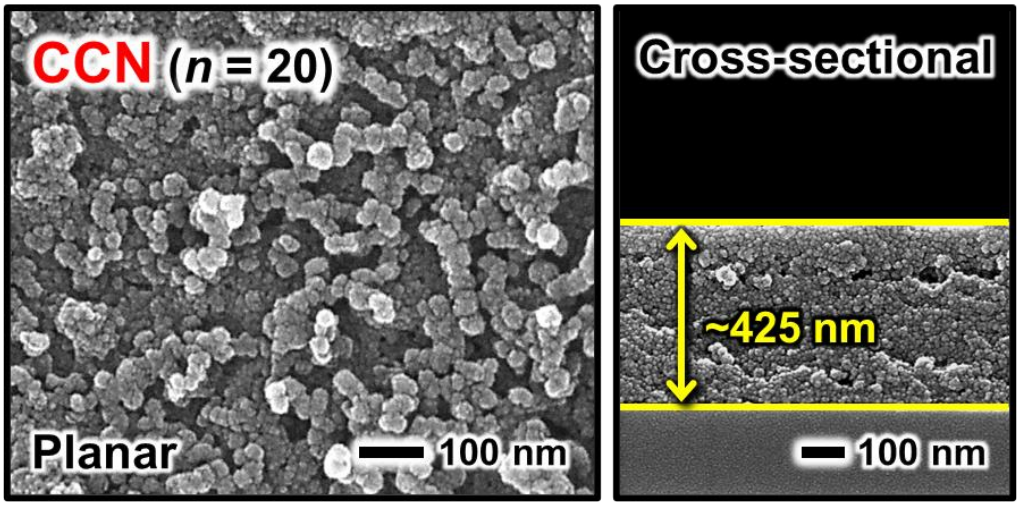

Figure S7. Planar and cross-sectional FE–SEM images of $(Fe_3O_4$ NP/CCN$)_{20}$ composites.

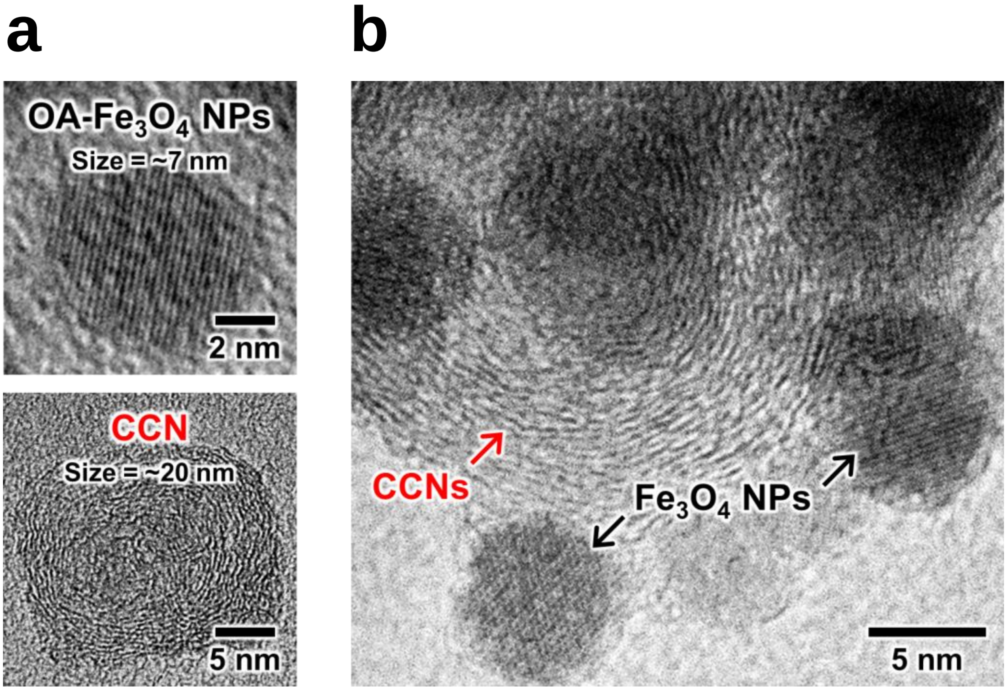

Figure S8. HR–TEM images of a) OA-Fe_3O_4 NPs with an average diameter of ~7 nm, CCN with an average particle size of ~20 nm, and b) (Fe_3O_4 NP/CCN)$_{20}$ composites.

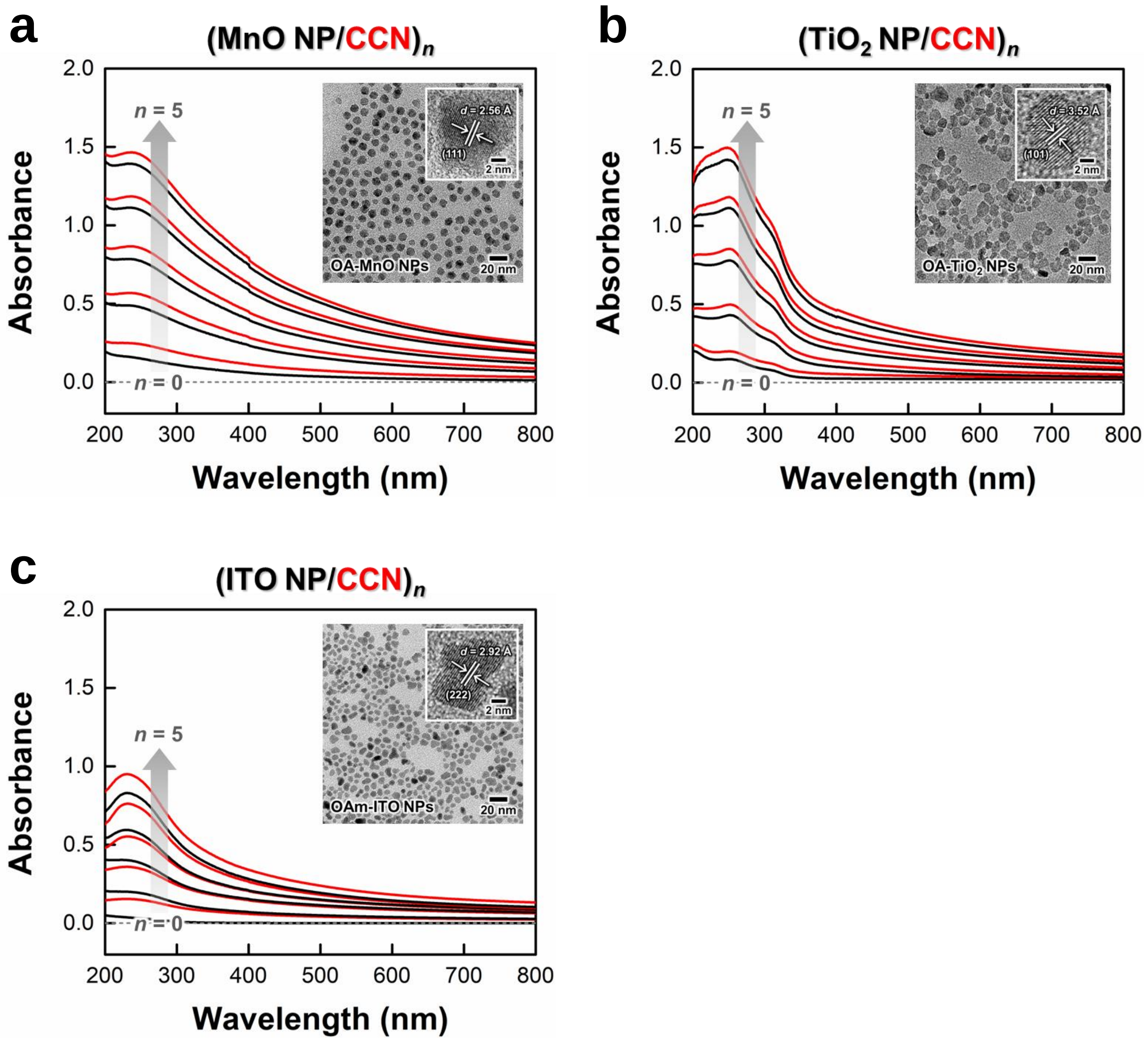

Figure S9. UV–vis absorbance spectra of (MO NP/CCN)$_n$ composites as a function of bilayer number (n) and HR–TEM images of MO NPs (inset): a) OA-MnO NPs, b) OA-TiO_2 NPs, c) OAm-ITO NPs. In this case, the abovementioned MO NPs were prepared according to the previously reported protocols.[S6–S8]

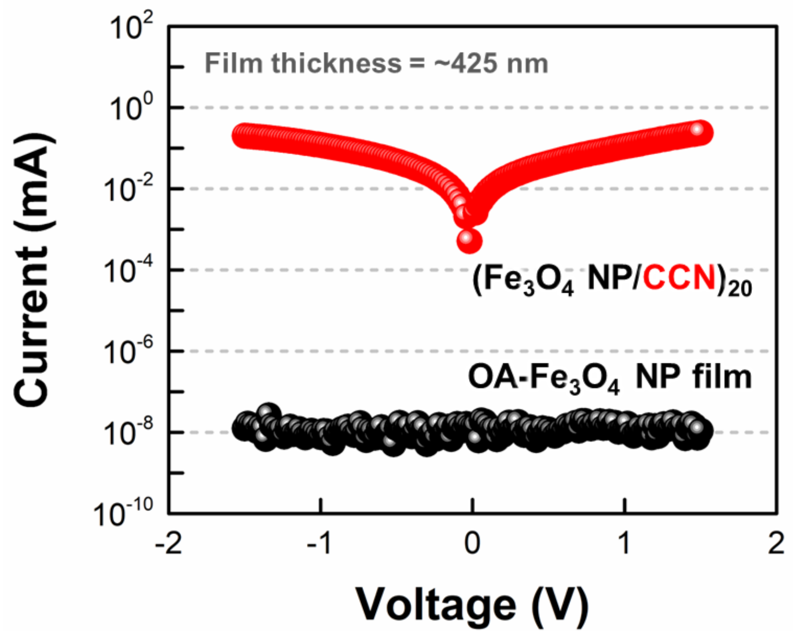

Figure S10. Current (log *I*)−voltage (*V*) curves of (Fe_3O_4 NP/CCN)$_{20}$ composites and OA-Fe_3O_4 NP films with the same film thickness (~425 nm) under the external voltage range of ±1.5 V.

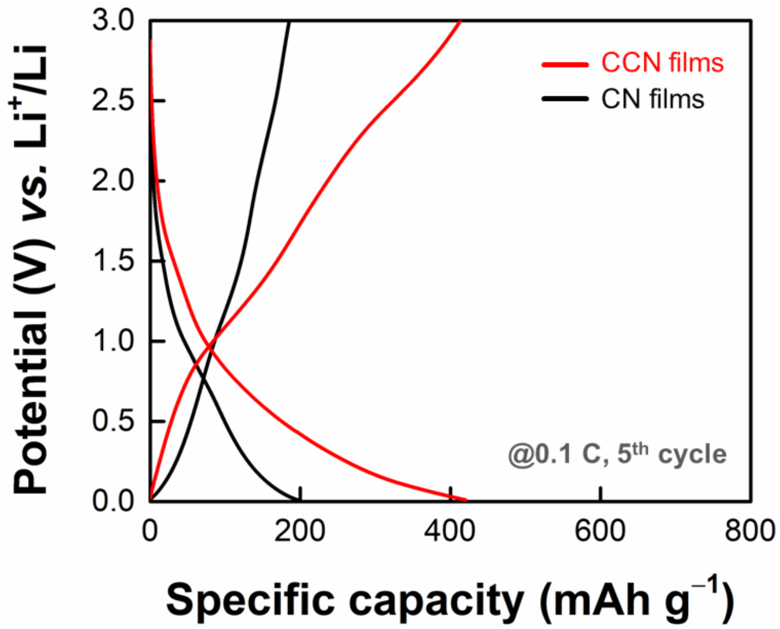

Figure S11. GCD profiles (5th cycle) of CCN films and non-functionalized CN films at 0.1 C. In this case, two different films were prepared through a spin-coating method using blended solutions, composed of carbon components (CCNs or CNs) and COOH-functionalized PAA binders with a weight ratio of 8:2.

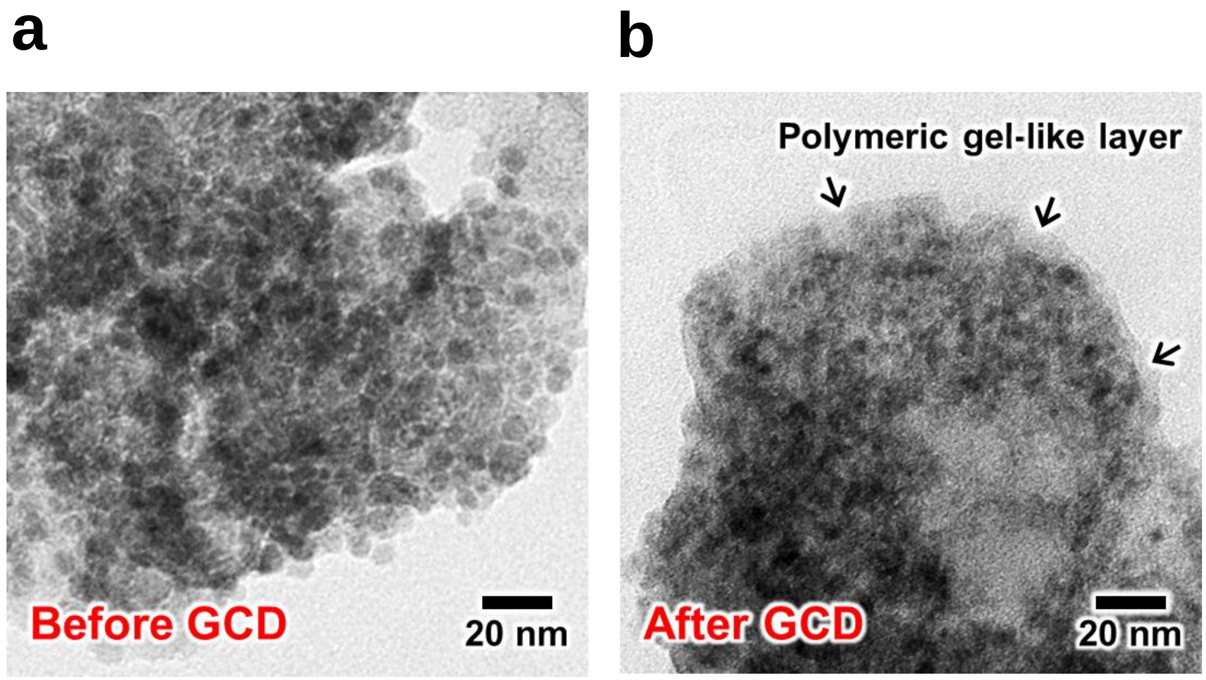

Figure S12. HR–TEM images of (Fe_3O_4 NP/CCN)$_{20}$-LIB electrodes a) before and b) after GCD cycles in the lithiation state.

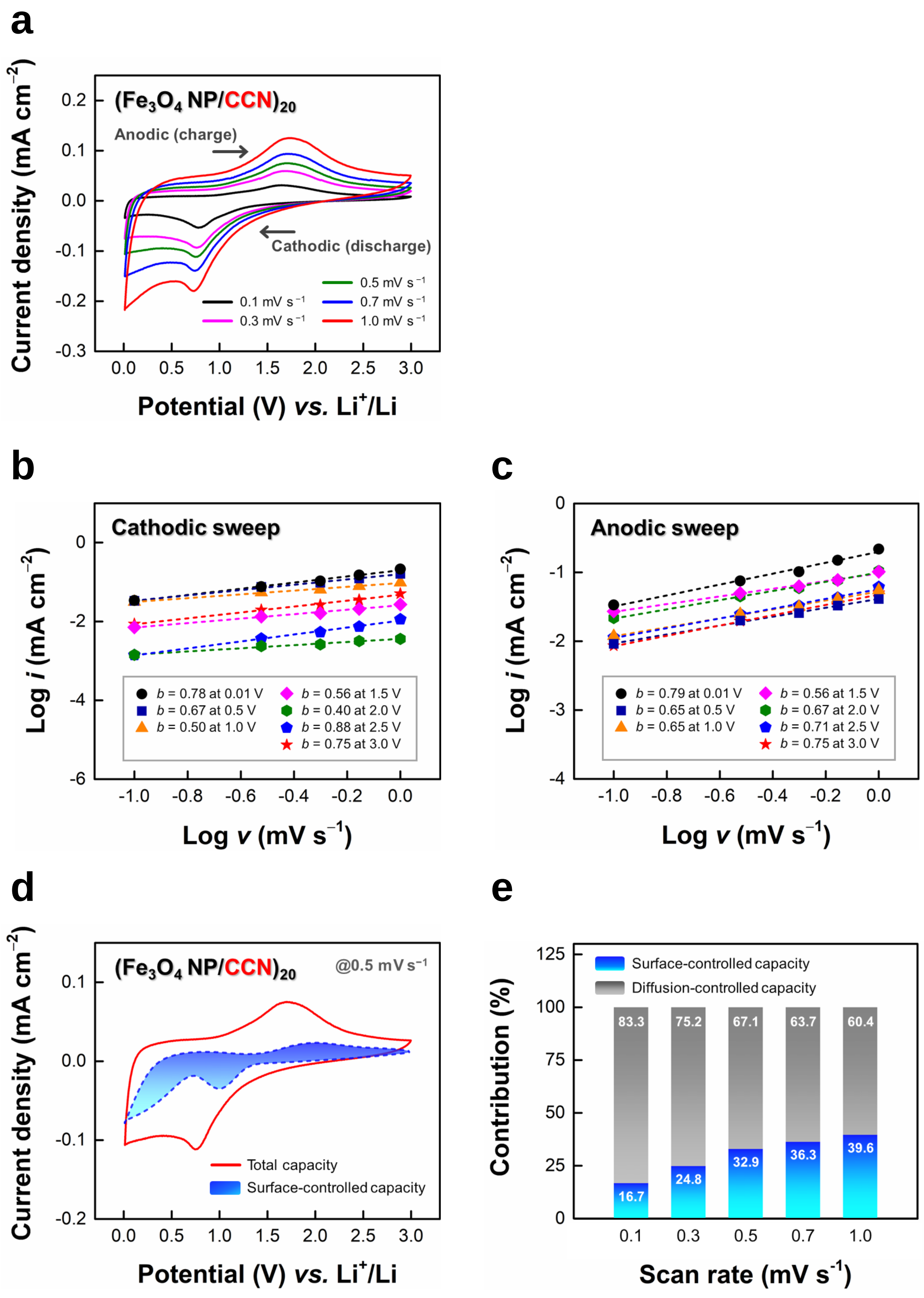

Figure S13. a) Scan rate-dependent CV curves (in the range of 0.1 to 1.0 mV s^{-1}) of (Fe_3O_4

NP/CCN)$_{20}$-LIB electrodes and the corresponding plots of log *i vs.* log *v* under b) cathodic and c) anodic sweeps. d) Separation of surface-controlled capacity from the total capacity of (Fe_3O_4 NP/CCN)$_{20}$-LIB electrodes at a scan rate of 0.5 mV s^{-1}. e) Contribution ratios of surface- and diffusion-controlled behaviors at different scan rates for (Fe_3O_4 NP/CCN)$_{20}$-LIB electrodes.

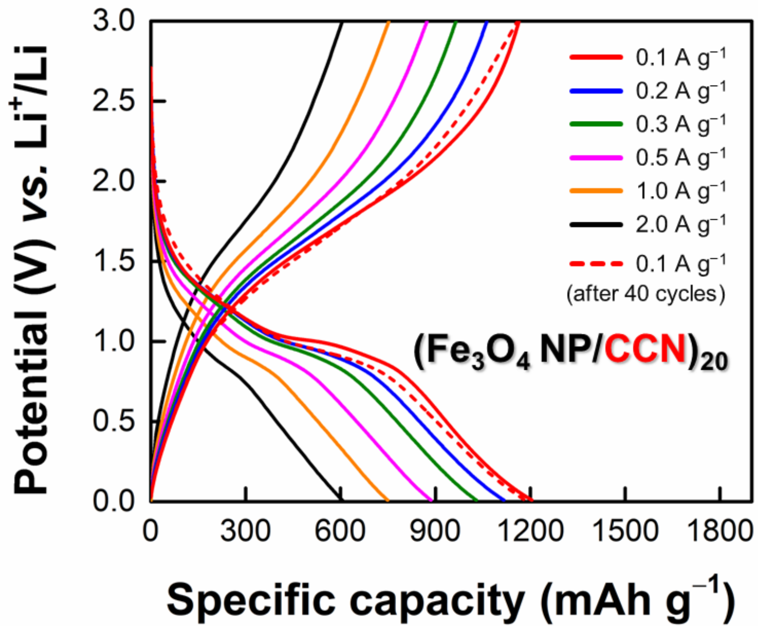

Figure S14. GCD profiles of (Fe_3O_4 NP/CCN)$_{20}$-LIB electrodes during cycling at different current densities of 0.1 (5th cycle), 0.2 (10th cycle), 0.3 (15th cycle), 0.5 (20th cycle), 1.0 (25th cycle), 2.0 (30th cycle), and 0.1 A g^{-1} (40th cycle).

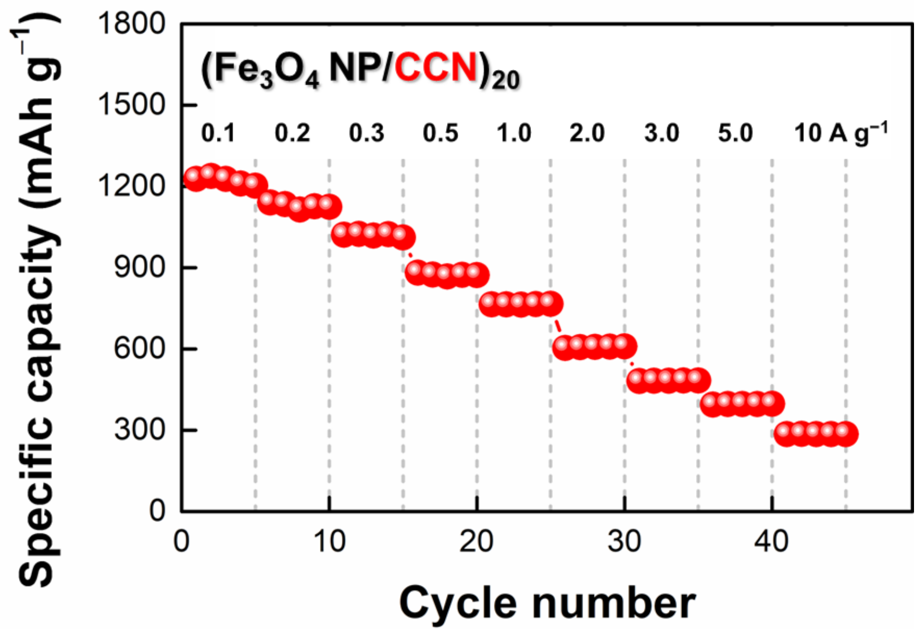

Figure S15. Rate capabilities of (Fe_3O_4 NP/CCN)$_{20}$-LIB electrodes at varied current densities from 0.1 to 10 A g^{-1} after five initial activation cycles (at 0.1 A g^{-1}).

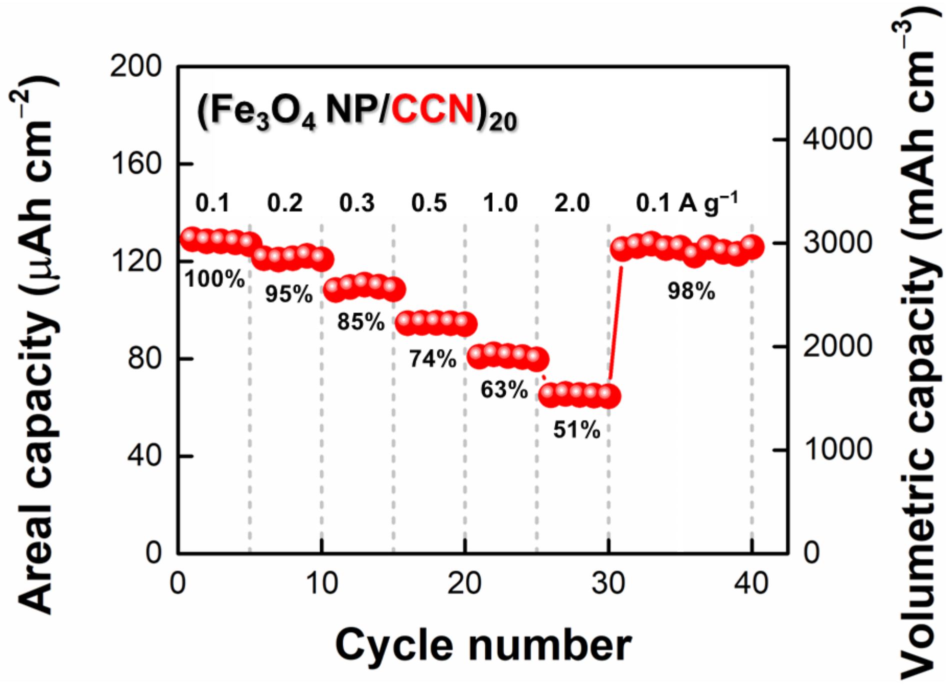

Figure S16. Current density-dependent areal (left axis) and volumetric (right axis) capacities of $(Fe_3O_4$ NP/CCN$)_{20}$-LIB electrodes in the range of 0.1 to 2.0 A g^{-1}.

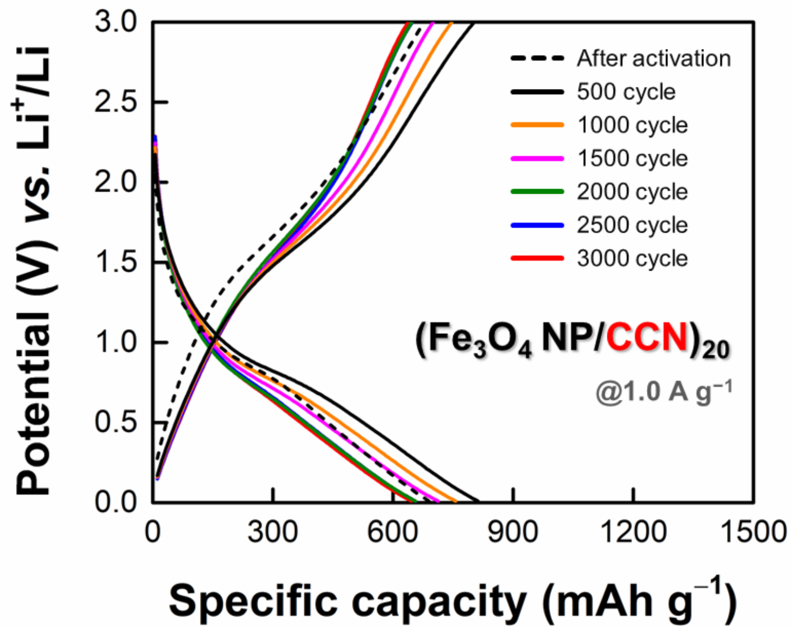

Figure S17. GCD profiles of (Fe_3O_4 NP/CCN)$_{20}$-LIB electrodes at selected cycles (500, 1000, 1500, 2000, 2500, and 3000 cycles) during cycling tests at a current density of 1.0 A g^{-1}.

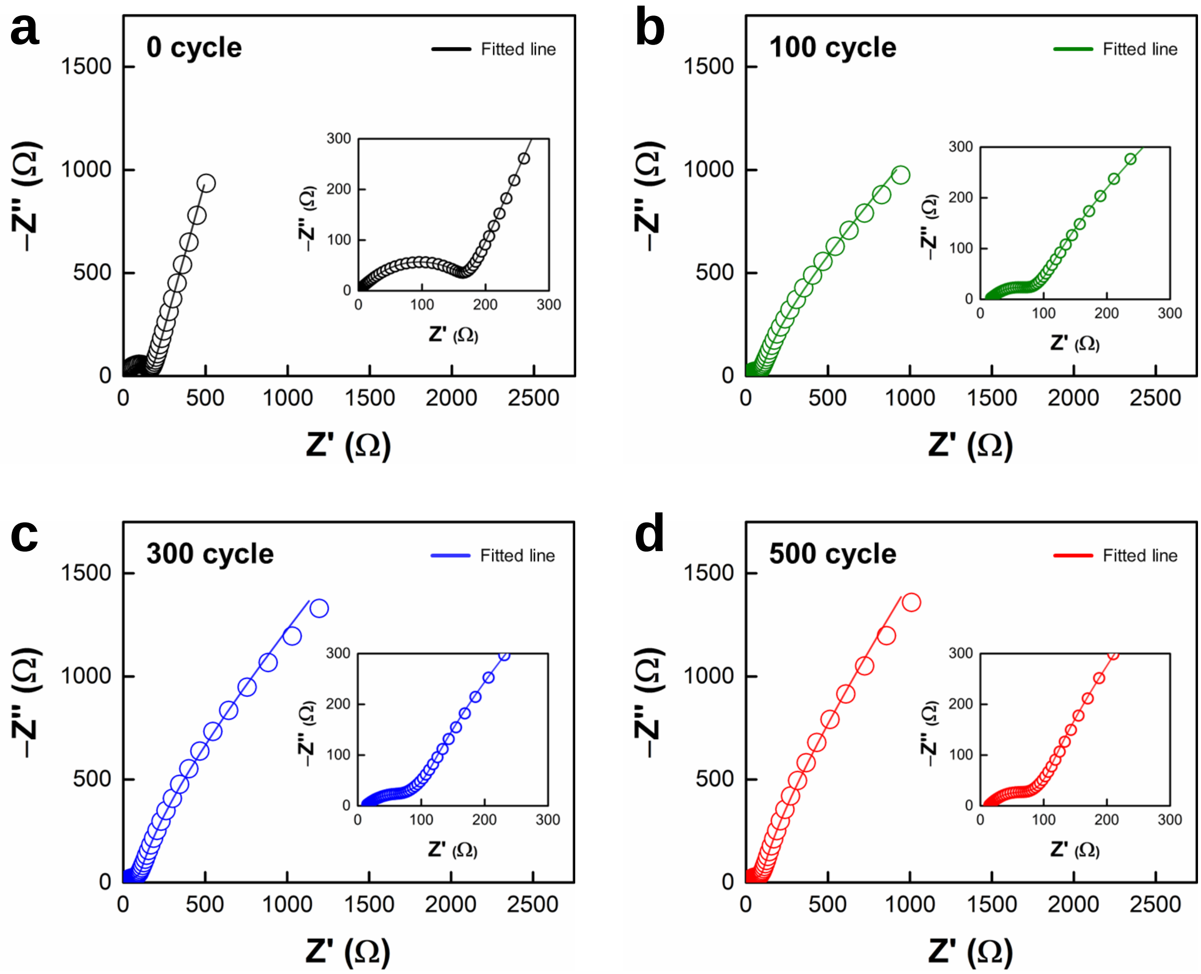

e

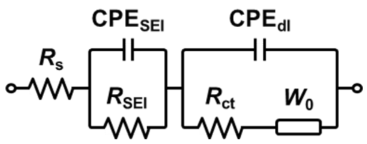

Parameter	0 cycle	100 cycle	300 cycle	500 cycle
R_s (Ω)	2.4	13.5	12.4	12.5
R_{SEI} (Ω)	10.8	22.7	22.3	21.4
R_{ct} (Ω)	161	67.7	67.4	66.9

Figure S18. Nyquist plots and the fitted lines of (Fe_3O_4 NP/CCN)$_{20}$-LIB electrodes during GCD tests at a current density of 1.0 A g^{-1} at a) 0, b) 100, c) 300, and d) 500 cycles. e) Simplified equivalent circuit and parameter values used to fit the experimental impedance spectra.

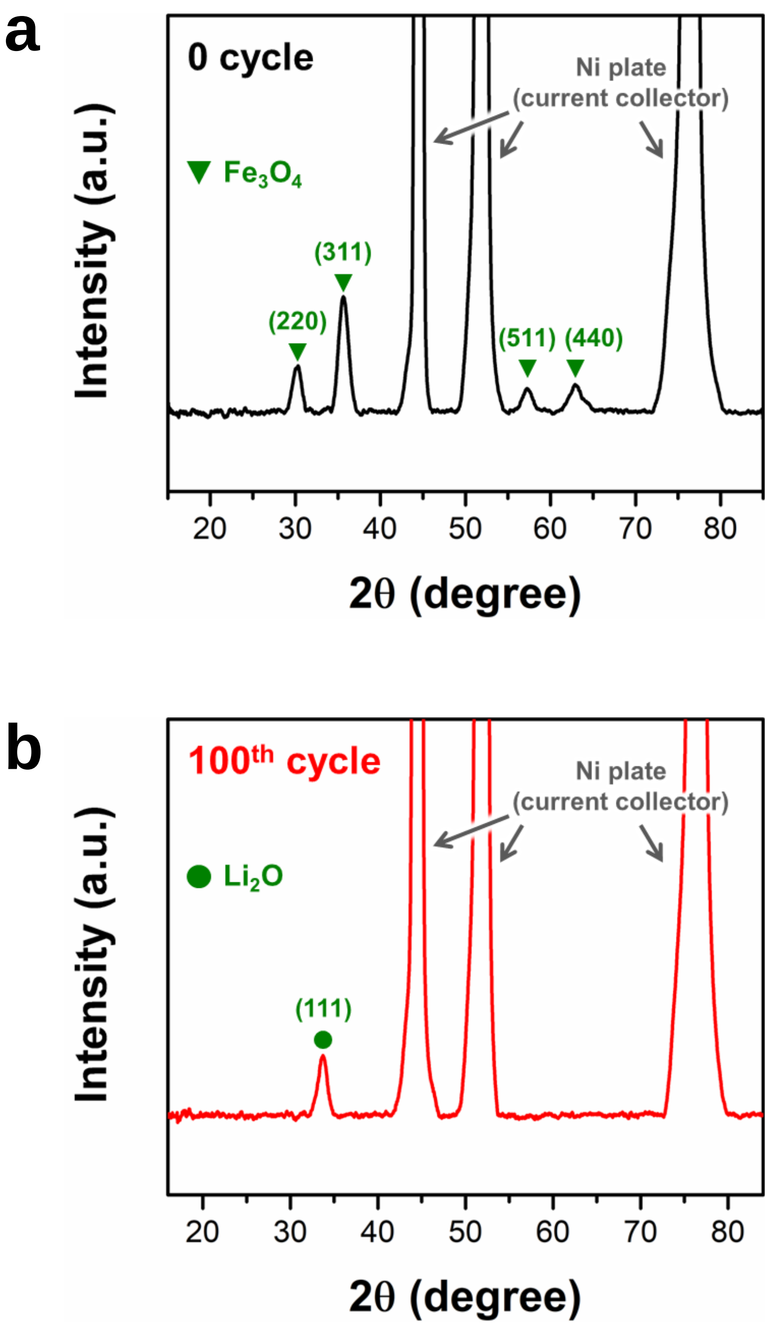

Figure S19. XRD patterns of (Fe_3O_4 NP/CCN)$_{20}$-LIB electrodes (based on Ni plates as current collectors) a) before and b) after 100 GCD cycles at a current density of 1.0 A g^{-1}.

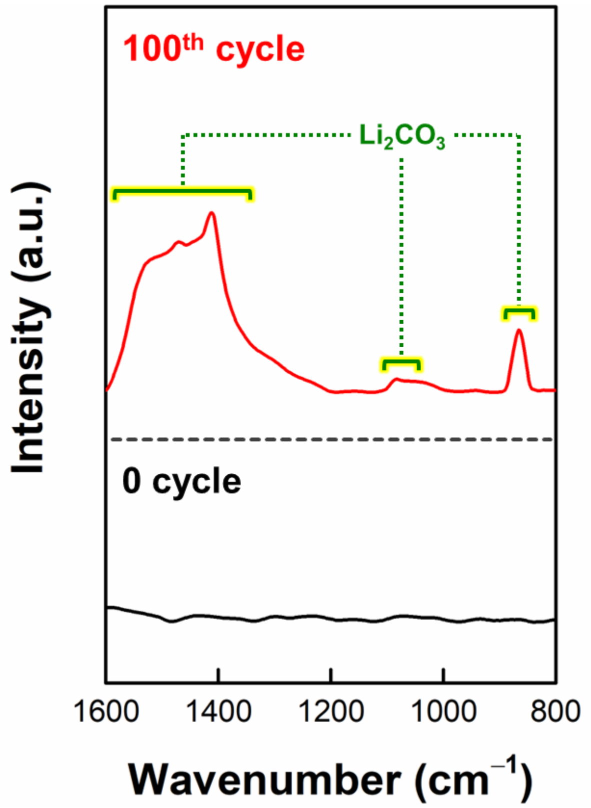

Figure S20. FTIR spectra of (Fe_3O_4 NP/CCN)$_{20}$-LIB electrodes before (bottom) and after 100 GCD cycles (top) at a current density of 1.0 A g^{-1}.

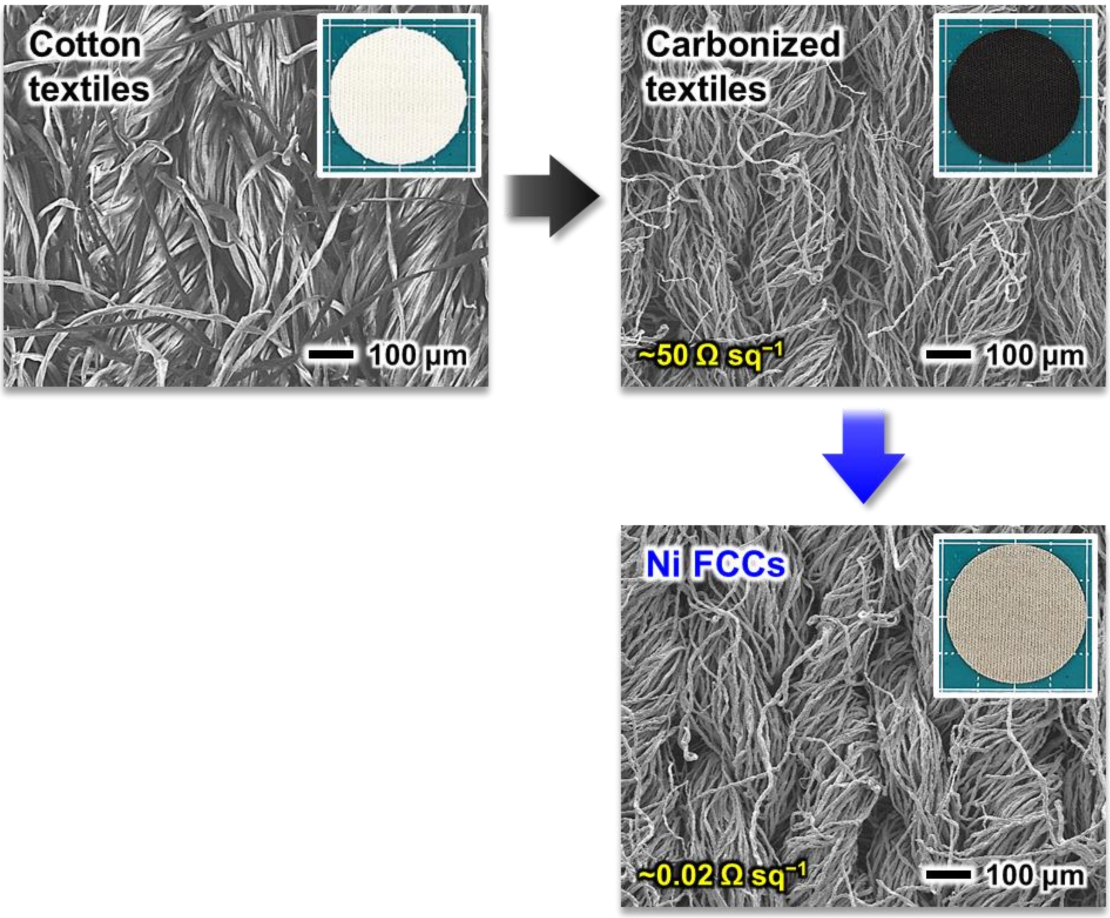

Figure S21. Planar FE–SEM images and photographs (insets) during the preparation of porous FCCs using the carbonization-assisted Ni electrodeposition of cotton textiles.

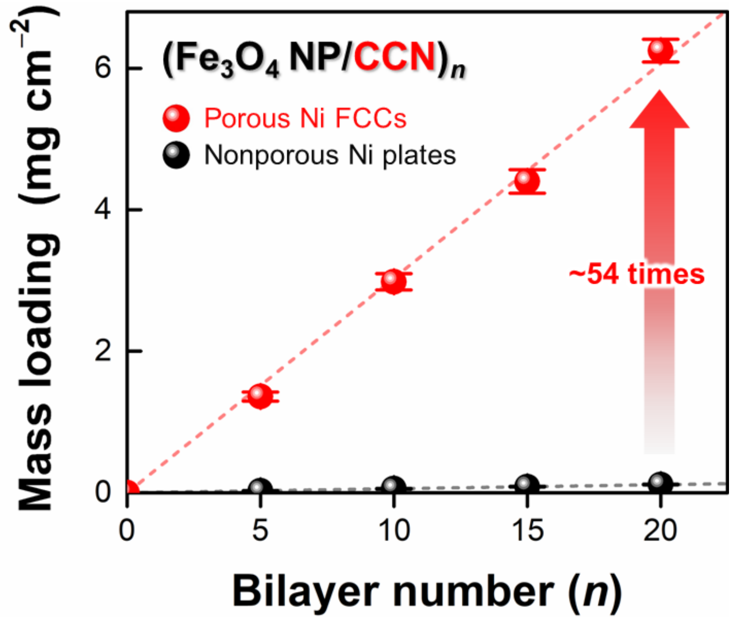

Figure S22. Comparison of the total mass loading of (Fe_3O_4 NP/CCN)$_n$ composites between on porous Ni FCCs and on nonporous Ni plates with increasing bilayer number (*n*) from 0 to 20.

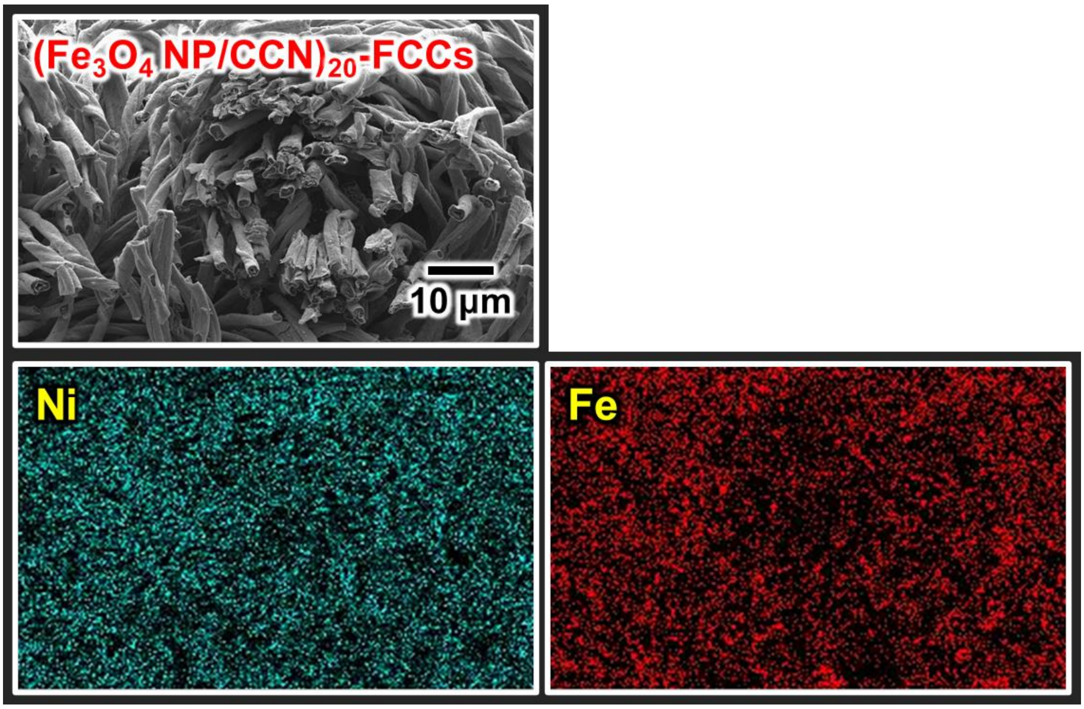

Figure S23. Cross-sectional FE–SEM and EDS elemental mapping images of (Fe_3O_4 NP/ CCN)$_{20}$-FCCs.

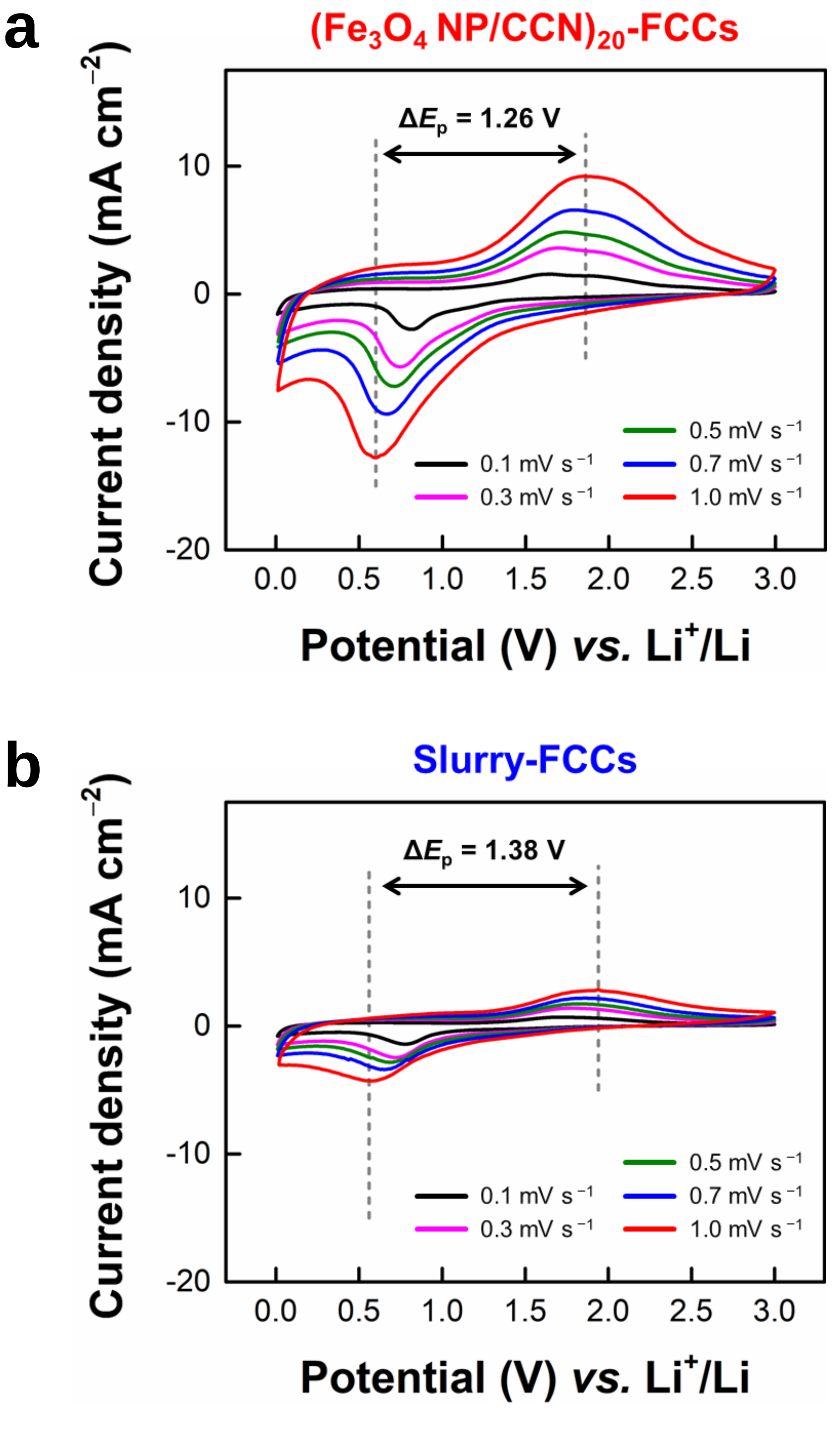

Figure S24. Scan rate-dependent CV curves of a) (Fe_3O_4 NP/CCN)$_{20}$-FCCs and b) slurry-FCCs at 0.1, 0.3, 0.5, 0.7, and 1.0 mV s^{-1}.

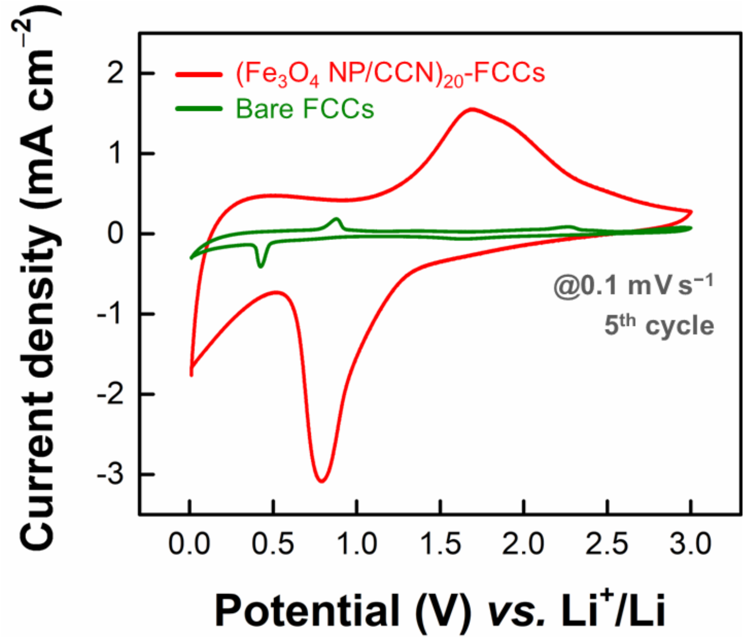

Figure S25. CV curves (5th cycle) of (Fe_3O_4 NP/CCN)$_{20}$-FCCs and bare Ni FCCs at a scan rate of 0.1 mV s^{-1}.

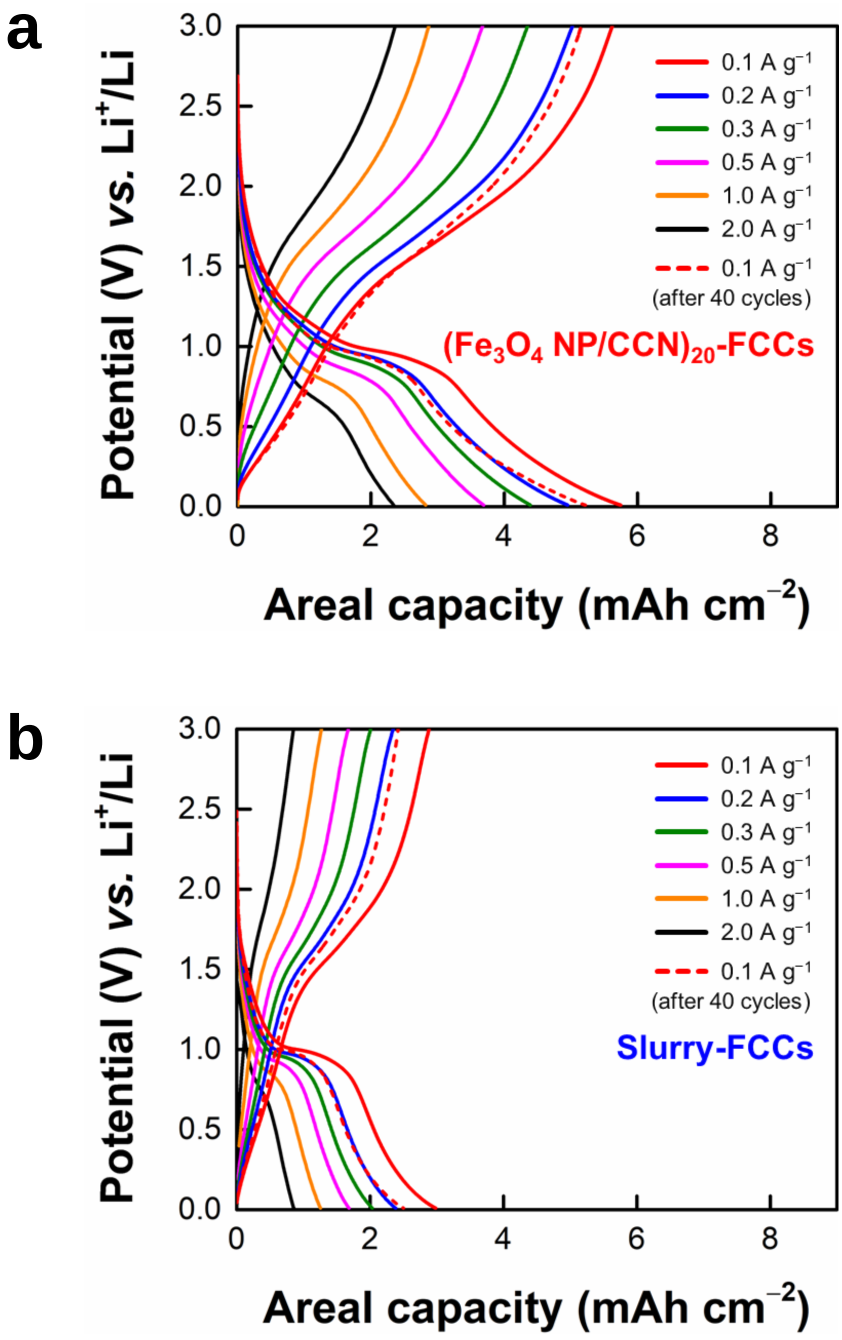

Figure S26. GCD profiles of a) (Fe_3O_4 NP/CCN)$_{20}$-FCCs and b) slurry-FCCs during cycling at different current densities of 0.1 (5th cycle), 0.2 (10th cycle), 0.3 (15th cycle), 0.5 (20th cycle), 1.0 (25th cycle), 2.0 (30th cycle), and 0.1 A g^{-1} (40th cycle).

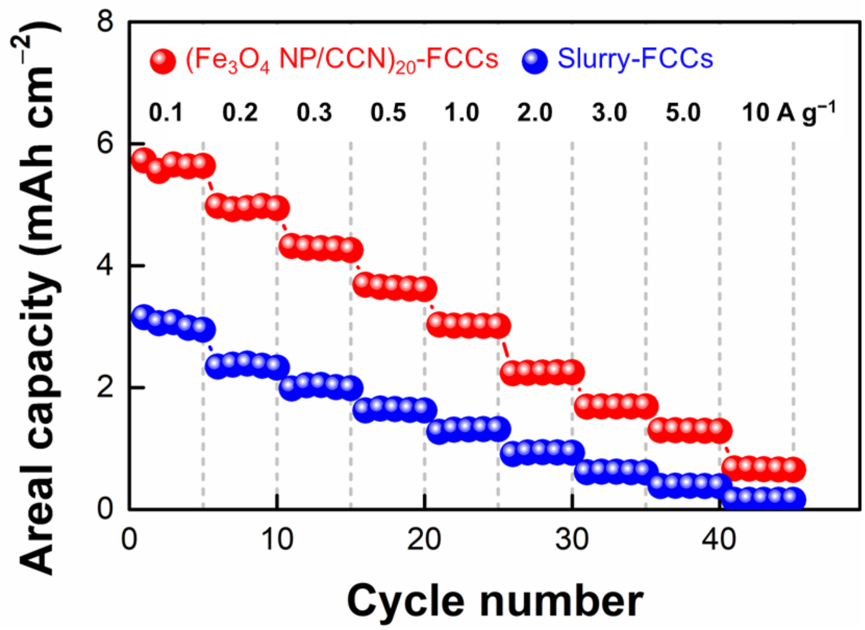

Figure S27. Comparison of rate capabilities between (Fe_3O_4 NP/CCN)$_{20}$-FCCs and slurry-FCCs at varied current densities from 0.1 to 10 A g^{-1} after five initial activation cycles (at 0.1 A g^{-1}). In this case, the (Fe_3O_4 NP/CCN)$_{20}$-FCCs exhibited a capacity retention of ~11.7% at 10 A g^{-1}, which was notably higher compared to the slurry-FCCs with a capacity retention of ~5.2%.

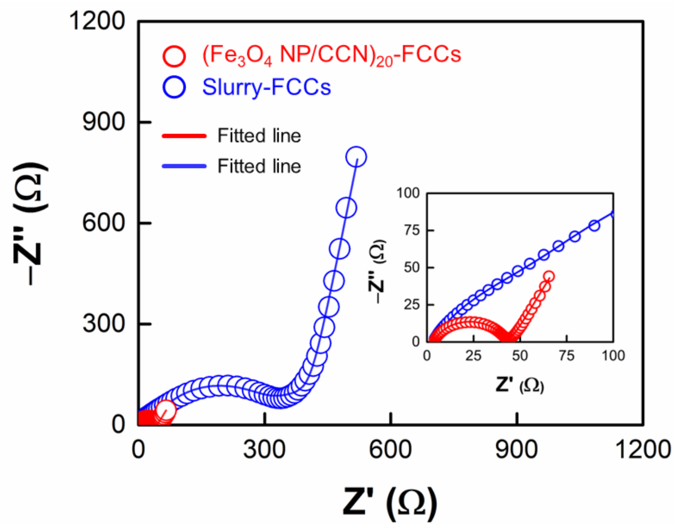

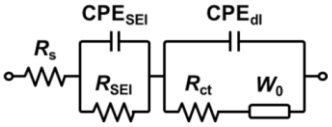

Parameter	(Fe_3O_4 NP/CCN)$_{20}$-FCCs	Slurry-FCCs
R_s (Ω)	4.1	4.2
R_{SEI} (Ω)	3.6	30.4
R_{ct} (Ω)	44	346

Figure S28. Nyquist plots and the fitted lines of (Fe_3O_4 NP/CCN)$_{20}$-FCCs and slurry-FCCs. The simplified equivalent circuit and parameter values used to fit the experimental impedance spectra are shown in the right panel.

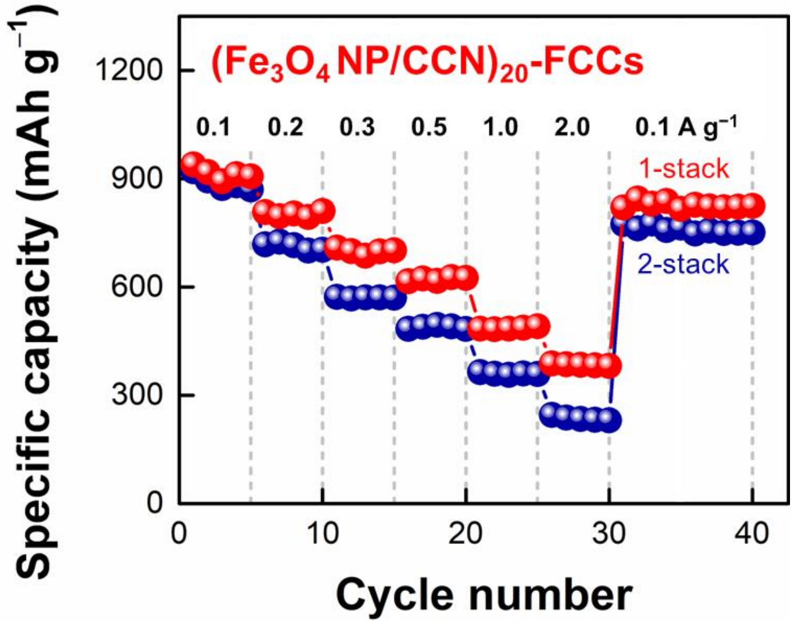

Figure S29. Specific capacities of multi-stacked (Fe_3O_4 NP/CCN)$_{20}$-FCCs with varying current density from 0.1 to 2.0 A g^{-1} after five initial activation cycles (at 0.1 A g^{-1}). In this case, the specific capacities are calculated based on the total mass loading of the composites (~6.2 mg cm^{-2} for 1-stack and ~12.4 mg cm^{-2} for 2-stack) on FCCs.

Table S1. Comparison of areal capacities of metal oxide-based 3D LIB anodes using porous current collectors.

Electrode materials	Substrate	Method	Total mass loading ($mg\ cm^{-2}$)	Areal capacity ($mAh\ cm^{-2}$)	Ref.
**Fe_3O_4 NPs /CCN**	**Ni-electroplated FCCs**	**CCN-mediated nanoblending assembly**	**6.2 (1-stack)**	**5.67 (at 0.1 A g^{-1})**	**Our work**
**Fe_3O_4 NPs /CCN**	**Ni-electroplated FCCs**	**CCN-mediated nanoblending assembly**	**12.4 (2-stack)**	**11.0 (at 0.1 A g^{-1})**	**Our work**
Fe_3O_4 @[a)]RGO	Carbon paper	Hydrothermal growth, electrostatic assembly, and thermal reduction	*0.47	*0.81 (0.5 A g^{-1})	[S9]
FeO_x nanoarrays	Copper foam	Hydrothermal growth	2.5	3.33 (at 0.25 A g^{-1})	[S10]
FeP@C nanotubes	Carbon fabric	Hydrothermal growth and phosphorization	1.8	1.73 (at 0.18 mA cm^{-2})	[S11]
$CoFe_2O_4$ nanowires	Carbon cloth	Hydrothermal growth	1.7-2.0	2.41 (at 0.5 A g^{-1})	[S12]
CoO nanowires	Carbon cloth	Hydrothermal growth	1.5	1.95 (at 0.1 A g^{-1})	[S13]
CoO /Co_3O_4	Ni foam	Hydrothermal growth and electrothermal waves	2.4	4.00 (at 0.5 mA cm^{-2})	[S14]
Zn/Co_2O_4 nanoplates	Carbon cloth	Solution growth and annealing	1.2	3.01 (at 0.24 mA cm^{-2})	[S15]

ZnO nanomembranes	Carbon foam	Immersion and pyrolysis	3.0-4.0	4.3 (at 0.08 A g^{-1})	[S16]
NiO@C nanosheets	Carbon cloth	Electrochemical deposition, hydrothermal growth, and ion exchange	4.0	3.08 (at 0.25 mA cm^{-2})	[S17]
$NiCo_2O_4$ nanowires	Carbon fabric	Hydrothermal growth	1.2	*1.23 (at -)	[S18]
SnO_2 nanosheet @[b)]AC	Carbon textile	Hydrothermal growth	3.2	*3.43 (at 0.05 A g^{-1})	[S19]

[a)](RGO: reduced graphene oxide); [b)](AC: amorphous carbon)

*Total mass loading and areal capacity values were estimated from the given data in the literatures.

Supplementary References

[S1] S. Sun, H. Zeng, D. B. Robinson, S. Raoux, P. M. Rice, S. X. Wang, G. Li, *J. Am. Chem. Soc.* **2004**, *126*, 273.

[S2] I. Cho, Y. Song, S. Cheong, Y. Kim, J. Cho, *Small* **2020**, *16*, 1906768.

[S3] V. Augustyn, P. Simon, B. Dunn, *Energy Environ. Sci.* **2014**, ***7***, 1597.

[S4] Y. Zeng, R. Hao, B. Xing, Y. Hou, Z. Xu, *Chem. Commun.* **2010**, *46*, 3920.

[S5] W. Bu, Z. Chen, F. Chen, J. Shi, *J. Phys. Chem. C* **2009**, *113*, 12176.

[S6] J. Park, K. An, Y. Hwang, J.-G. Park, H.-J. Noh, J.-Y. Kim, J.-H. Park, N.-M. Hwang, T. Hyeon, *Nat. Mater.* **2004**, *3*, 891.

[S7] D. Pan, N. Zhao, Q. Wang, S. Jiang, X. Ji, L. An, *Adv. Mater.* **2005**, *17*, 1991.

[S8] M. Kanehara, H. Koike, T. Yoshinaga, T. Teranishi, *J. Am. Chem. Soc.* **2009**, *131*, 17736.

[S9] T. Gao, C. Xu, R. Li, R. Zhang, B. Wang, X. Jiang, M. Hu, Y. Bando, D. Kong, P. Dai, X.-B. Wang, *ACS Nano* **2019**, *13*, 11901.

[S10] F. Zhang, C. Yang, H. Guan, Y. Hu, C. Jin, H. Zhou, L. Qi, *ACS Appl. Energy Mater.* **2018**, *1*, 5417.

[S11] X. Xu, J. Liu, Z. Liu, Z. Wang, R. Hu, J. Liu, L. Ouyang, M. Zhu, *Small* **2018**, *14*, 1800793.

[S12] S. Zhao, J. Guo, F. Jiang, Q. Su, G. Du, *Mater. Res. Bull.* **2016**, *79*, 22.

[S13] S. Zhao, J. Guo, F. Jiang, Q. Su, J. Zhang, G. Du, *J. Alloys Compd.* **2016**, *655*, 372.

[S14] W. Kim, D. Shin, B. Seo, S. Chae, E. Jo, W. Choi, *ACS Nano* **2022**, *16*, 17313.

[S15] T. Liu, W. Wang, M. Yi, Q. Chen, C. Xu, D. Cai, H. Zhan, *Chem. Eng. J.* **2018**, *354*, 454.

[S16] Y. Zhao, G. Huang, Y. Li, R. Edy, P. Gao, H. Tang, Z. Bao, Y. Mei, *J. Mater. Chem. A* **2018**, *6*, 7227.

[S17] S. Chen, R. Tao, J. Tu, P. Guo, G. Yang, W. Wang, J. Liang, S.-Y. Lu, *Adv. Funct. Mater.* **2021**, *31*, 2101199.

[S18] J.-M. Son, S. Oh, S.-H. Bae, S. Nam, I.-K. Oh, *Adv. Energy Mater.* **2019**, *9*, 1900477.

[S19] X. Min, B. Sun, S. Chen, M. Fang, X. Wu, Y. Liu, A. Abdelkader, Z. Huang, T. Liu, K. Xi, R. V. Kumar, *Energy Storage Mater.* **2019**, *16*, 597.
